# Spoken sentence comprehension in Mandarin-English bilinguals: a case against the universal processing advantage of subject-relatives

**DOI:** 10.3389/flang.2025.1703230

**Published:** 2026-02-02

**Authors:** Preeti Rishi, Yusheng Wang, Tracy Love, Henrike K. Blumenfeld

**Affiliations:** 1 Joint Doctoral Program in Language and Communicative Disorders, San Diego State University/University of California San Diego, San Diego, CA, United States; 2 School of Speech, Language, and Hearing Sciences, San Diego State University, San Diego, CA, United States

**Keywords:** bilingual, canonicity, language processing, Mandarin, relative clause, sentence comprehension, syntax

## Abstract

**Introduction:**

This study investigates sentence comprehension in Mandarin-English bilinguals, focusing on whether the widely reported, yet contested, subject-relative processing advantage extends to bilingual speakers. We evaluate which theoretical accounts, based on syntactic structure and canonicity, best explain cross-linguistic patterns of sentence processing.

**Methods:**

Using a sentence-picture matching task, we examined the comprehension of canonical (e.g., actives) and non-canonical (e.g., passives) sentence structures in English and Mandarin for bilingual speakers of varying ages and Mandarin and English proficiency levels across two separate studies (*n* = 18 and *n* = 35).

**Results:**

In English, bilingual participants exhibited a robust canonical sentence advantage across studies, with better comprehension of subject-relative over object-relative sentences and active over passive sentences, mirroring monolingual processing patterns. However, in Mandarin, comprehension patterns were less robust and more variable. While subject-relative and object-relative comprehension did not significantly differ at the group level, passive vs. active sentences consistently posed greater difficulty and increased performance variability across both studies, particularly among lower-performing individuals.

**Discussion:**

These results suggest that sentence comprehension is shaped by language-specific constraints rather than a universal subject-relative advantage. Findings align with unified theoretical accounts that incorporate canonicity-based and structural factors, including word order, syntactic structure, and experience-, usage-, and frequency-based influences. Our results highlight the complex interplay between the aforementioned factors that differ across languages, with implications for both theoretical linguistics and clinical applications.

## 1 Introduction

The process of language comprehension is intricate and involves the integration of information across several linguistic levels to allow listeners to extract a speaker’s meaning. At the sentence level, comprehension involves the interplay between lexical, syntactic, semantic, pragmatic, and discourse structure factors (Gibson and Pearlmutter, 1998; Tanenhaus and Trueswell, 1995) and these processing dynamics have been shown to differ across languages, allowing for identification of both universal and language-specific principles (e.g., Bates et al., 2001). Several theories capture the complexity of sentence comprehension, and each theory makes its own predictions for what types of sentences may be more or less difficult to comprehend cross-linguistically (e.g., O’Grady, 1997, 2011; Gibson, 1998; Mitchell et al., 1995; Bever, 1970). Sentence processing theories are based on a variety of linguistic, cognitive, and real-world factors that may differ across sentence types cross-linguistically, such as syntactic structure, memory constraints, frequency, prominence, and canonicity (typical word order in a given language). These theories are often supported by behavioral evidence from monolingual individuals cross-linguistically that includes accuracy rates, reaction times, and reading times for a variety of sentence constructions that vary in syntactic structure and canonicity (e.g., English: Gibson, 1998; Gibson et al., 2005; Gordon et al., 2002; Grodner and Gibson, 2005; King and Just, 1991; King and Kutas, 1995; MacWhinney, 1982; Stromswold et al., 1996; Traxler et al., 2002; Wanner and Maratsos, 1978; Dutch: Frazier, 1987; Mak et al., 2002, 2006; German: Mecklinger et al., 1995; Schriefers et al., 1995; Spanish: del Río et al., 2012; Sánchez et al., 2019; Italian: Domenico and Matteo, 2009; French: Holmes and O’Regan, 1981; Basque: Carreiras et al., 2010; Japanese: Ishizuka et al., 2006).

Two languages that have been studied more widely to test various sentence processing theories are English and Mandarin, where a majority of these studies have focused on monolingual speakers of each language (e.g., Chen et al., 2008; Gibson et al., 2005; Gibson and Wu, 2013; Hsiao and Gibson, 2003; Lin and Bever, 2006; Lin and Garnsey, 2011; Packard et al., 2011; Qiao et al., 2012; Sung et al., 2016; Traxler et al., 2002; Vasishth et al., 2013; Xu et al., 2019). English and Mandarin provide a unique language pair to test sentence processing theories because they generally share similar subject-verb-object word order yet use a different approach to relative clause formation (explained in Section 1.1 below). Here, we focus on investigating sentence comprehension in bilingual speakers of English and Mandarin. Given the lack of sentence processing theories for bilinguals, and the likelihood of overlapping processing principles across native and second languages, this study applies monolingual models to bilingual speakers to identify which model(s) best describe their processing.

### 1.1 Sentence constructions across English and Mandarin

English and Mandarin share several word-order and syntactic headedness properties, yet they also differ in important ways. The syntactic head of a phrase determines its category and structure; for example, in *eat the apple*, *eat* is the head, making it a verb phrase. In terms of syntactic headedness, English is a head-initial language while Mandarin is a typologically unique language with mixed-headedness. Similar to English, Mandarin verb phrases are generally head-initial (verb + object). Noun phrases, however, are exclusively head-final (modifier + noun; Li and Thompson, 1989). With respect to word order, English follows a strict subject-verb-object (SVO) word order where canonical sentences (sentences with typical word order) involve the agent (the doer of the action) followed by the patient (the entity that undergoes the action; see 1a below). Sentences in which patients precede agents are considered to have non-canonical (or atypical) word order in English (see 1b). However, Mandarin is less strict regarding word order and shares features of both SVO and SOV languages such as having (i) both VO and OV constructions; (ii) prepositions (SVO typical) and postpositions (SOV typical); (iii) auxiliaries that precede verbs (SVO typical); and (iv) relative clauses that precede the head noun (SOV typical; see Li and Thompson, 1989; Huang et al., 2009). Modern Mandarin, a relatively recent variety of Sinitic language, exhibits less flexibility in word order compared to Classical Chinese or other Chinese dialects. In most cases, Mandarin follows an SVO word order (Li and Thompson, 1989; Huang et al., 2009), where the agent precedes the patient, as such, we consider canonical word order in Mandarin to be like English with agent preceding patient (see 2a) and non-canonical word order with patient preceding agent (see 2b).


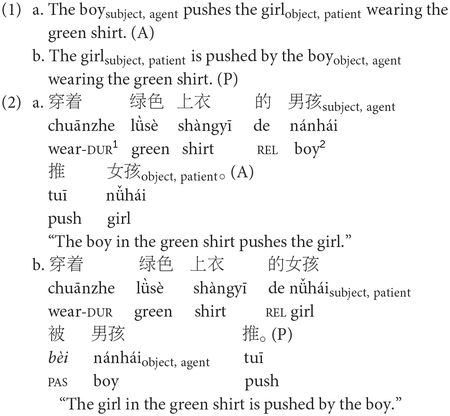


Sentence constructions that are often used to test various sentence processing theories include basic active (A) and passive (P) constructions and more complex subject-relative (SR) and object-relative (OR) clause sentences, given their differences in agent-patient ordering. Active sentences follow a canonical word order in English (see 1a above) and Mandarin (see 2a above) where the subject is the agent who performs the action. Passive sentences follow a non-canonical word order in English (see 1b above) and Mandarin (see 2b above) where the patient who receives the action is fronted to the subject position. Active and passive constructions allow for testing the reversal of agent and patient roles (Bever, 1970; Ferreira et al., 2002). Unlike active and passive constructions, the subject-object order within relative clauses sentences differs across English and Mandarin. A relative clause is a subordinate clause that provides additional information about a noun or pronoun, and begins with *who*, *which*, or *that* (see 3, 4,^3^ where SRC = subject-relative clause, ORC = object-relative clause). Relative clauses are classified as subject- or object-relative based on the syntactic role of the head noun they modify. In subject-relative clauses, the subject is modified (see 3a, 4a); in object-relative clauses, the object is modified (see 3b, 4b). For example, in sentence 3a, the relative clause *that chases the girl* gives further information about the head noun and subject *the boy*. In English relative clauses, the head noun comes before the clause, making them head-initial (SVO typical). The head noun originally appears as an argument within the relative clause but then moves to the left side of the structure. When it moves, it leaves behind a silent placeholder, often described as a trace (*t*_*i*_), a gap, or an empty argument position^4^ (see 3a, b; Government and Binding Theory; Chomsky, 1993). In contrast, Mandarin relative clauses are head-final (SOV typical), with the clause preceding the head noun and the trace appearing earlier (see 4a, b). Importantly, English and Mandarin subject- and object-relative clauses differ in canonicity. English subject-relatives follow canonical agent-before-patient order, whereas object-relatives are non-canonical. In contrast, Mandarin subject-relatives are non-canonical, and object-relatives are canonical. These constructions are useful for cross-linguistic sentence processing studies, as they use the same words but differ in order and structure, allowing researchers to isolate structural and word order effects.


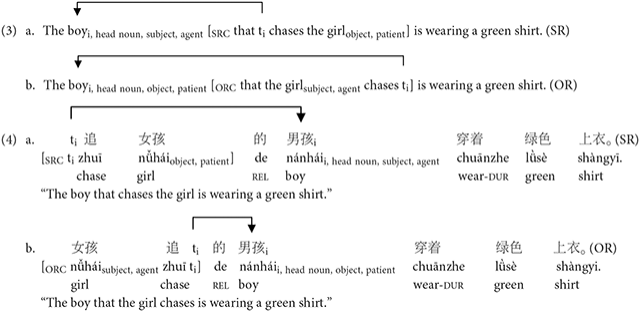


### 1.2 Previous cross-linguistic findings of sentence processing

Cross-linguistically, the consistency of sentence-processing findings for relative clause constructions varies systematically depending on whether a language employs head-initial or head-final relative clauses. For languages that use head-initial relative clauses, previous literature has found a processing advantage for subject-relative sentences using accuracy, reading time, or response time measures across several languages including English (Gibson, 1998; Gibson et al., 2005; Gordon et al., 2002; Grodner and Gibson, 2005; King and Just, 1991; King and Kutas, 1995; MacWhinney, 1982; Stromswold et al., 1996; Traxler et al., 2002; Wanner and Maratsos, 1978), Dutch (Frazier, 1987; Mak et al., 2002, 2006), German (Mecklinger et al., 1995; Schriefers et al., 1995), Spanish (del Río et al., 2012; Sánchez et al., 2019), Italian (Domenico and Matteo, 2009), and French (Holmes and O’Regan, 1981). When examining head-final relative clause processing, the results become much less consistent in confirming a universal subject-relative processing advantage. In fact, evidence against a subject-relative processing advantage has been found in languages such as Mandarin (Chen et al., 2008; Gibson and Wu, 2013; Hsiao and Gibson, 2003; Lin and Garnsey, 2011; Packard et al., 2011; Qiao et al., 2012; Sung et al., 2016; Xu et al., 2019), Basque (Carreiras et al., 2010), and Japanese (Ishizuka et al., 2006), whose relative clauses are all head-final. However, within Mandarin specifically, findings remain conflicting where some studies maintain a socalled universal advantage in subject-relative clause processing (Lin and Bever, 2006; Vasishth et al., 2013). Vasishth et al. (2013) conducted a meta-analysis of 15 previous relative clause studies in Mandarin and found that the subject-relative preference was more dominant than the object-relative preference. They also attempted to replicate prior findings through three self-paced reading experiments, two of which showed a subject-relative advantage while one showed an object-relative advantage, reflecting the same inconsistency seen in the broader literature. We return to this discrepancy in Section 4. For a review and meta-analysis on the debated subject-relative advantage across languages, see Lau and Tanaka (2021) and Tanaka et al. (2024), respectively.

Beyond relative clause processing, past research has also examined sentence comprehension more broadly by comparing canonical and non-canonical sentence structures, including actives, passives, and relative clauses constructions, in both healthy and clinical populations. In healthy adult English speakers, non-canonical passive and object-relative constructions were found to be frequently misinterpreted as compared to canonical active and subject-relative constructions (Ferreira, 2003). Neuroimaging studies have likewise revealed that non-canonical sentence constructions elicit greater left inferior frontal cortex and left supramarginal gyrus activation, which is thought to reflect increased memory demands needed to re-analyze the sentence constituents for correct understanding (Bahlmann et al., 2007). Processing of canonical and non-canonical constructions has also been widely studied in clinical populations, including adults with aphasia (acquired language disorder frequently due to stroke or brain injury) and children with Developmental Language Disorder (DLD). Across these clinical populations cross-linguistically, non-canonical sentences generally appear to be harder to process regardless of the headedness of the language, as typically evidenced by lower accuracy rates (English aphasia: Cho-Reyes and Thompson, 2012; Horne et al., 2022; Love and Oster, 2002; Meyer et al., 2012; Pettigrew and Hillis, 2014; Chinese aphasia: Law and Leung, 1998, 2000; Su et al., 2007; Wang and Thompson, 2016; Dutch aphasia: Bastiaanse and Edwards, 2004; Italian aphasia: Barbieri et al., 2013; English developmental language disorder: Montgomery and Evans, 2009).

### 1.3 Theoretical accounts for sentence processing

A wide range of sentence processing theories has been proposed to explain how individuals interpret structural relationships in language, each offering different predictions across languages. For the present study, and in line with our experimental manipulations, we focus on two prominent and contrasting accounts, structural-based and top-down canonicity-based approaches, which generate different predictions for English and Mandarin sentence processing in healthy adults. Our stimuli were designed to vary primarily in syntactic structure and word-order canonicity, and therefore our theoretical focus is anchored to these dimensions rather than to factors we did not manipulate, such as corpus-based distributions or individual usage patterns. At the same time, we recognize that canonicity extends beyond word order alone. Broader usage-based perspectives emphasize that canonicity reflects not only structural heuristics but also frequency, experience, and patterns of language use (e.g., Mitchell et al., 1995; Bybee, 2006; Goldberg, 2006; Ellis et al., 2016). Although these dimensions are conceptually relevant to sentence processing, they were not directly examined in the present study. Finally, while additional processing theories exist (see Table 1 for an overview), their predictions largely align with one of the two focal accounts considered here and thus fall outside the scope of our primary analyses.

#### 1.3.1 Structural-based accounts

**FIGURE 1:**
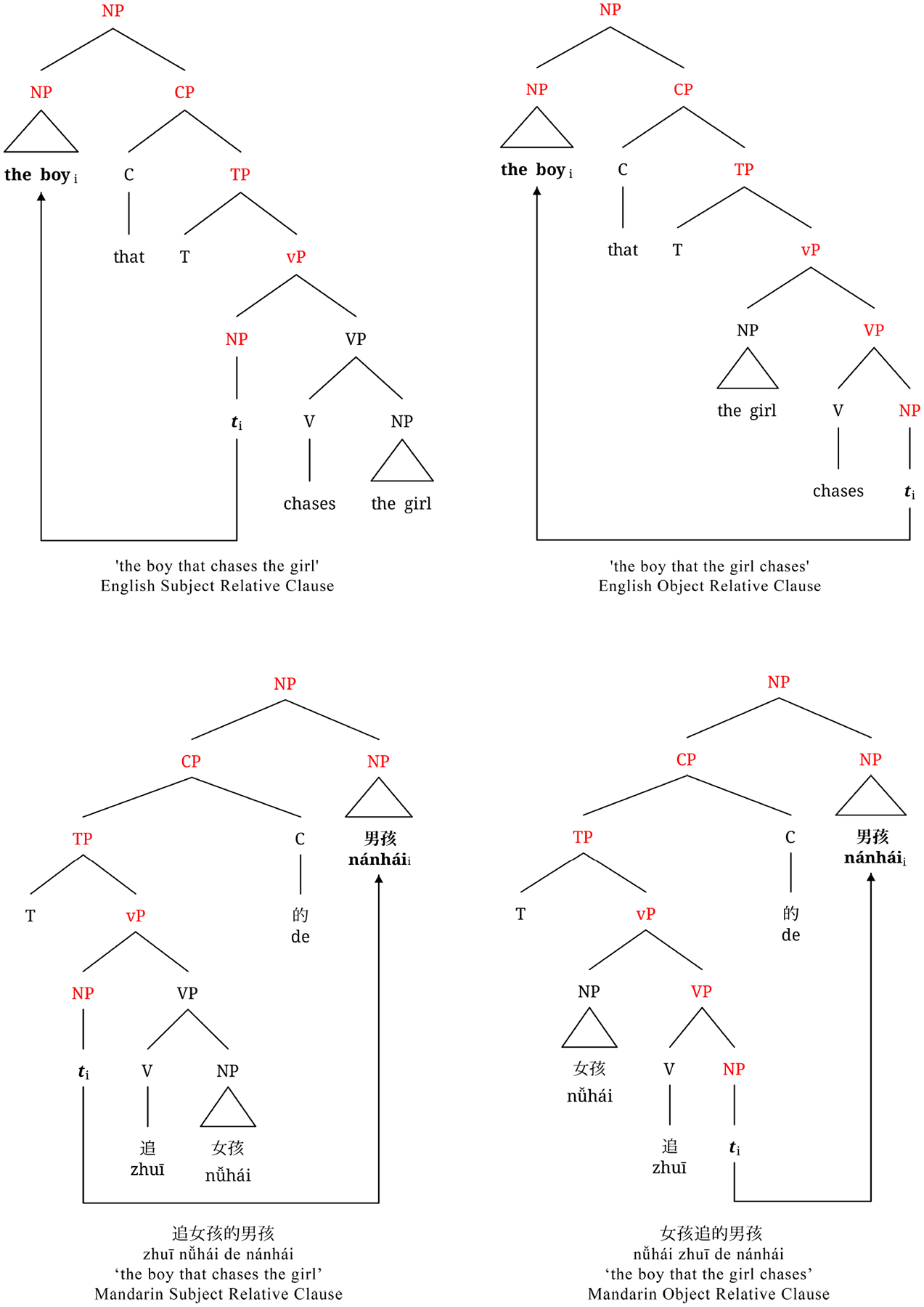
Syntactic structure of English subject- and object-relative clauses **(top)** and Mandarin subject- and object-relative clauses **(bottom)**. Intervening nodes between the head noun/filler and gap/trace are colored using red text.

A primary structural-based account is the structural distance account (O’Grady, 1997) which states that as the structural distance, defined as the number of intervening syntactic nodes, increases between the head noun and the trace, sentence complexity also increases. Relative clause constructions easily illustrate the structural distance account (see Figure 1; intervening nodes are colored with red text). As shown in Figure 1, an English object-relative clause (top right) has seven intervening nodes between the head noun “the boy_i_” and trace *t*_*i*_ as compared to six intervening nodes in a subject-relative clause (top left), rendering the subject-relative clause less complex and easier to process. For Mandarin, an object-relative clause (bottom right) has more intervening nodes between the head noun 男孩/*nánhái* and trace *t*_*i*_ as compared to the subject-relative clause (bottom left), resulting in the same prediction as in English. Thus, the structural distance account predicts a uniform processing preference for subject-relative clauses cross-linguistically. In other words, extracting from an object position requires traversing more nodes, and thus, this object position is always less accessible for extraction than the subject position according to Keenan and Comrie’s (1977) Accessibility Hierarchy.

Structural-based accounts fit the consistent subject-relative processing advantage found via increased response accuracy, faster reading times, etc. across head-initial relative clauses in languages such as English, Dutch, French, and German, but remain contested in languages with head-final relative clause constructions such as Mandarin, Basque, and Japanese, where results have been mixed. Structural accounts can also be applied to simple active and passive sentences, where passive constructions likewise have intervening nodes between the head noun and trace, while active sentences do not have any such trace or intervening nodes. As such, passive constructions are seen as more complex according to the structural distance account across English and Mandarin. Other well-known theories, such as frequency, experience, usage-based accounts (e.g., Mitchell et al., 1995; Bybee, 2006; Goldberg, 2006; Ellis et al., 2016) and prominence-based accounts (e.g., Mak et al., 2006; O’Grady, 2011; Lin, 2018), make similar predictions as structural-based accounts (see Table 1).

#### 1.3.2 Top-down canonicity-based accounts

One top-down account of sentence-processing is the top-down heuristic account for sentence processing (Bever, 1970). This theory considers the top-down factor of canonical thematic patterns in sentences where the language processor is employing a canonical sentence schema to interpret sentences (Swinney and Love, 1998). According to Bever’s account, syntactic processing involves expectations where the language users’ experience and usage with the language influences sentence parsing. In English and Mandarin, the canonical thematic pattern is agent-verb-patient. In English, active sentences and subject-relative constructions follow this dominant canonical thematic pattern while passive sentences and object-relative constructions do not. In Mandarin, however, active sentences and *object*-relative constructions follow the dominant canonical agent-verb-patient thematic pattern while subject-relative constructions do not. Thus, top-down canonicity-based processing models would predict a processing advantage for English active and subject-relative constructions compared to passive and object-relative constructions. In Mandarin, this account would predict an advantage for processing active and *object*-relative constructions compared to passive and subject-relative constructions.

Importantly, canonicity reflects not only word order processing heuristics but also frequency, usage, and experience-based properties of language (e.g., Mitchell et al., 1995; Bybee, 2006; Goldberg, 2006; Ellis et al., 2016). Canonical mappings are not only structurally preferred but also tend to be more frequent and semantically prototypical in natural language use. From this perspective, the observed advantage for canonical sentences may arise from the interaction between *structural efficiency* and *experiential entrenchment*, that is, frequent exposure to familiar sentence types increases ease of processing (MacDonald, 2013). Accordingly, we recognize canonicity as both a structural and experience-based phenomenon. Interestingly, in Mandarin, structurally non-canonical subject-relative sentences tend to be more frequent in corpus data than their canonical object-relative counterparts (Pu, 2007), meaning that canonicity yields mixed predictions for Mandarin relative clause processing where structural components of canonicity and frequency/experience/usage-based components of canonicity point in opposite directions. For other accounts that make similar predictions as top-down canonicity models across English and Mandarin, see Table 1 (e.g., Gibson, 1998, 2000).

### 1.4 The current studies

Given the many competing factors that shape Mandarin sentence processing, and the resulting conflicting findings in the literature, this area of research warrants further examination. The motivation behind the current studies is to build on the literature dominated by single-language findings in monolinguals and further investigate the processing of simple and complex sentences across the two languages of Mandarin-English bilinguals to determine which theory or theories of sentence processing can best account for sentence comprehension findings in bilinguals. Examination of the subject-relative advantage through the lens of bilingualism allows direct within-subjects cross-linguistic comparison that controls for the between-subjects variability that may cloud cross-linguistic comparison of monolinguals’ performance. Moreover, this approach can speak to the broader question of how language-specific constraints interact with cognitive processing mechanisms during sentence processing. We include four sentence types—actives, passives, subject-relatives, and object-relatives—where actives and passives form a critical pair and subject-relatives and object-relatives form another critical pair. Within these pairs, each sentence differs in syntactic structure and canonicity (word order), allowing us to test our two sentence processing theories of interest, structural-based and top-down canonicity-based accounts.

For the current studies, Mandarin-English bilinguals may be predicted to perform differently than monolingual Mandarin or English speakers given cross-linguistic competition and/or cooperation (e.g., Bates and MacWhinney, 1982; Bates et al., 2001). However, according to Román and Gómez-Gómez’s (2022) systematic review and meta-analysis, first language processing in bilinguals is subject to only small transformations, if any, from a second language. As such, we are curious if bilingual Mandarin-English speakers will demonstrate a converging pattern of sentence processing across both of their languages or if participants will show diverging sentence processing patterns across languages. The former result would offer support for structural-based theories. The latter result would provide convincing evidence in favor of top-down canonicity-based accounts, especially since the divergence of sentence processing across Mandarin and English would hold true in a single individual regardless of cross-linguistic influence between the bilingual speaker’s languages. Our research aims are to investigate the contested universal subject-relative processing advantage in Mandarin-English bilingual speakers and determine which theory or theories of sentence processing best support sentence processing cross-linguistically across simple and complex sentences in bilingual speakers.

Our corresponding research questions are as follows:

- Do Mandarin-English bilinguals demonstrate similar sentence processing patterns across Mandarin and English, suggesting convergence consistent with structure-based theories?
- Alternatively, do bilinguals exhibit language-specific processing patterns (e.g., subject-relative advantages in English and object-relative advantages in Mandarin), consistent with top-down canonicity-based accounts?

We predict that if *structural-based* accounts best reflect cross-linguistic performance, then participants’ performance should pattern the same way across English and Mandarin, with higher accuracy on subject-relatives compared to object-relatives and actives compared to passives, resulting in a universal subject-relative processing advantage. On the other hand, if *top-down canonicity* accounts best capture cross-linguistic sentence comprehension, then we would expect the opposite results that defy a universal subject-relative advantage, namely that participants perform more strongly for canonical constructions: object-relatives in Mandarin and subject-relatives in English and actives in both.

In Study 1, we present data from Mandarin-dominant individuals who listened to sentences and matched the sentence’s meaning to one of three pictures that required identification of the correct agent, patient, and action of the sentence. The task included active, passive, subject-relative, and object-relative sentences across English and Mandarin. For Study 2, we extend the findings of Study 1 across a larger age range of Mandarin-English bilinguals with varying proficiency levels to ensure that our findings from Study 1 were not due to age-related issues, limited English abilities, nor high Mandarin proficiency. While syntactic canonicity effects have been found in younger and older adults (e.g., Love and Oster, 2002), some studies have suggested that these effects may become more pronounced in older adults (Wingfield et al., 2006; Peelle et al., 2010). Further, as the primary focus of the current paper was to better understand canonicity effects in Mandarin, with potential convergence or divergence with patterns in English, a group of early and highly proficient speakers of Mandarin who were late learners of English and older adults were recruited for Study 1, with a wider range of ages and language profiles considered in Study 2.

## 2 Study 1: sentence processing in Mandarin-dominant bilingual elders

Study 1 involves older (*M*_age_ = 76.8, *SD* = 4.08) bilinguals that are dominant in Mandarin, with both higher self-reported and objective proficiency in Mandarin as well as greater exposure to Mandarin as compared to English (see Sections 2.1 and 2.3.2, Table 2).

### 2.1 Participants

Thirty-two Mandarin-English bilingual older adults were recruited from a local community center in Southern California via announcements and flyers. Ten individuals did not complete the study in its entirety, three individuals reported a history of neural trauma, and one individual scored over 1.5 standard deviations below the typical average on the *Montreal Cognitive Assessment* for their age range (*MoCA*; Nasreddine et al., 2005; Yu et al., 2012; Hong et al., 2022; described in Section 2.2.1) and were therefore all excluded. Thus, Study 1 included a final sample of 18 participants^5^ (*M*_*age*_ = 76.8, *SD* = 4.08, range: 70–86; 10 female, eight male).

This study was approved by and carried out in accordance with the recommendations of San Diego State University’s Institutional Review Board. Written informed consent was obtained from all participants in accordance with the Declaration of Helsinki. All participants passed basic vision and hearing screeners indicating that vision and hearing (with corrections such as eyeglasses or hearing aids, as necessary) were within appropriate limits to complete Study 1. Two participants reported a history of learning disability, but outcomes of all analyses remained the same when these two participants were omitted, so both participants were included in the current cohort. All participants reported immigrating to the United States later in life, with a mean age of immigration of 68.6 years old (*SD* = 4.98, range: 61–76). Participants reported Mandarin as their L1 and English as an L2 or beyond, where dialects of Chinese were considered to each be their own language when assigning the order of language acquisition. Other languages that were acquired by participants included Russian (*n* = 13), Tianjing dialect (*n* = 1), Shanghainese dialect (*n* = 2), Wuhan dialect (*n* = 1), and Japanese (*n* = 1). All participants were Mandarin dominant (*M* = 0.47, *SD* = 0.19; on a scale from −1 to 1 where positive numbers indicate being Mandarin-dominant) as calculated across self-report and objective language performance (see Section 2.3.2). Moreover, on average, participants reported higher proficiency in and exposure to Mandarin (proficiency: *M* = 8.29, *SD* = 0.97; out of 10; exposure: *M* = 88.8%, *SD* = 9.57) compared to English (proficiency: *M* = 2.12, *SD* = 1.74; out of 10; exposure: *M* = 11.21%, *SD* = 9.93) on the *Language Experience and Proficiency Questionnaire* (*LEAP-Q*; Blumenfeld et al., 2017; Marian et al., 2007) and demonstrated stronger Mandarin verbal fluency abilities (*M*_raw_ score = 16.3; *SD* = 3.54) as compared to English (*M*_raw score_ = 8.47; *SD* = 5.18; all *p*’s < 0.001, see Section 2.2.1). Table 2 presents a summary of participants’ linguistic and cognitive profiles; we address these factors in the Sections 2.5 and 4. All participants were offered compensation for their participation in the study.

### 2.2 Procedure, materials and experimental design

After informed consent, participants were administered the following relevant tasks across five sessions: (1) a Mandarin interview version of the *LEAP-Q* (Blumenfeld et al., 2017; Marian et al., 2007); (2) English and Mandarin verbal fluency tasks; (3) the Beijing version of the *MoCA* (*MoCA-BJ*; Nasreddine et al., 2005; Yu et al., 2012); and (4) the English version of the *SOAP* (*Subject-relative, Object-relative, Active, Passive*) *Syntactic Battery of Sentence Comprehension* (*E-SOAP*; Love and Oster, 2002), a picture-matching test used to probe sentence comprehension abilities, as well as a Mandarin version of the battery developed for Study 1 (*M-SOAP*). Tasks were administered individually by trained Mandarin-English bilingual researchers in a quiet testing room at the local community center where participants had been recruited. Mandarin and English tasks were administered in separate sessions, with Mandarin presented first to familiarize participants with the task format and to minimize the need for translation during the English sessions given that Mandarin was their dominant language. Participants completed two sessions in Mandarin followed by three in English (with only minimal switching to Mandarin as needed), for a total of five sessions. Session data were audio recorded, and tasks were scored during the sessions using physical scoresheets with reliability checks in place (described in Section 2.3.1).

#### 2.2.1 Language history, proficiency, and cognitive background measures

Detailed information on participants’ language history and proficiency was obtained using a Mandarin structured oral interview version of the *LEAP-Q* (Blumenfeld et al., 2017; Marian et al., 2007) administered verbally and in print by a trained Mandarin-speaking research assistant (see Table 2). Participants completed English and Mandarin animal and grocery verbal fluency tasks, given that verbal fluency performance based on semantic category cues has been shown to index language proficiency in bilinguals (e.g., Blumenfeld et al., 2016; Gollan et al., 2002). Animal and grocery categories were chosen since (a) animals are a commonly used verbal fluency cue (e.g., Rosselli et al., 2000; Portocarrero et al., 2007; Bialystok et al., 2008); and (b) the grocery cue (listing items that can be purchased) indexed everyday language use (e.g., Clark et al., 2009). Participants were verbally instructed to name as many items within each category as they could within 60 s without repetition. The *MoCA-BJ* (Nasreddine et al., 2005; Yu et al., 2012) was administered in Mandarin, the participants’ native and dominant language, to gauge participants’ cognitive abilities. The *MoCA* is a well-established cognitive screening tool for older adults.

#### 2.2.2 English SOAP syntactic battery of sentence comprehension

The *SOAP* syntactic battery (Love and Oster, 2002) consists of 40 experimental sentences that target reversible actions with active (A), passive (P), subject-relative (SR), and object-relative (OR) constructions (10 each). Sample English *SOAP* (*E-SOAP*) sentences are found in 5a–d, and all the *E-SOAP* sentences can be found in Appendix 1.

(5) a. (A) The girl chases the small boy in the green shirt.
  b. (P) The boy is chased by the girl in the green shirt.
  c. (SR) The boy that chases the girl is wearing a green shirt.
  d. (OR) The boy that the girl chases is wearing a green shirt.

**FIGURE 2:**
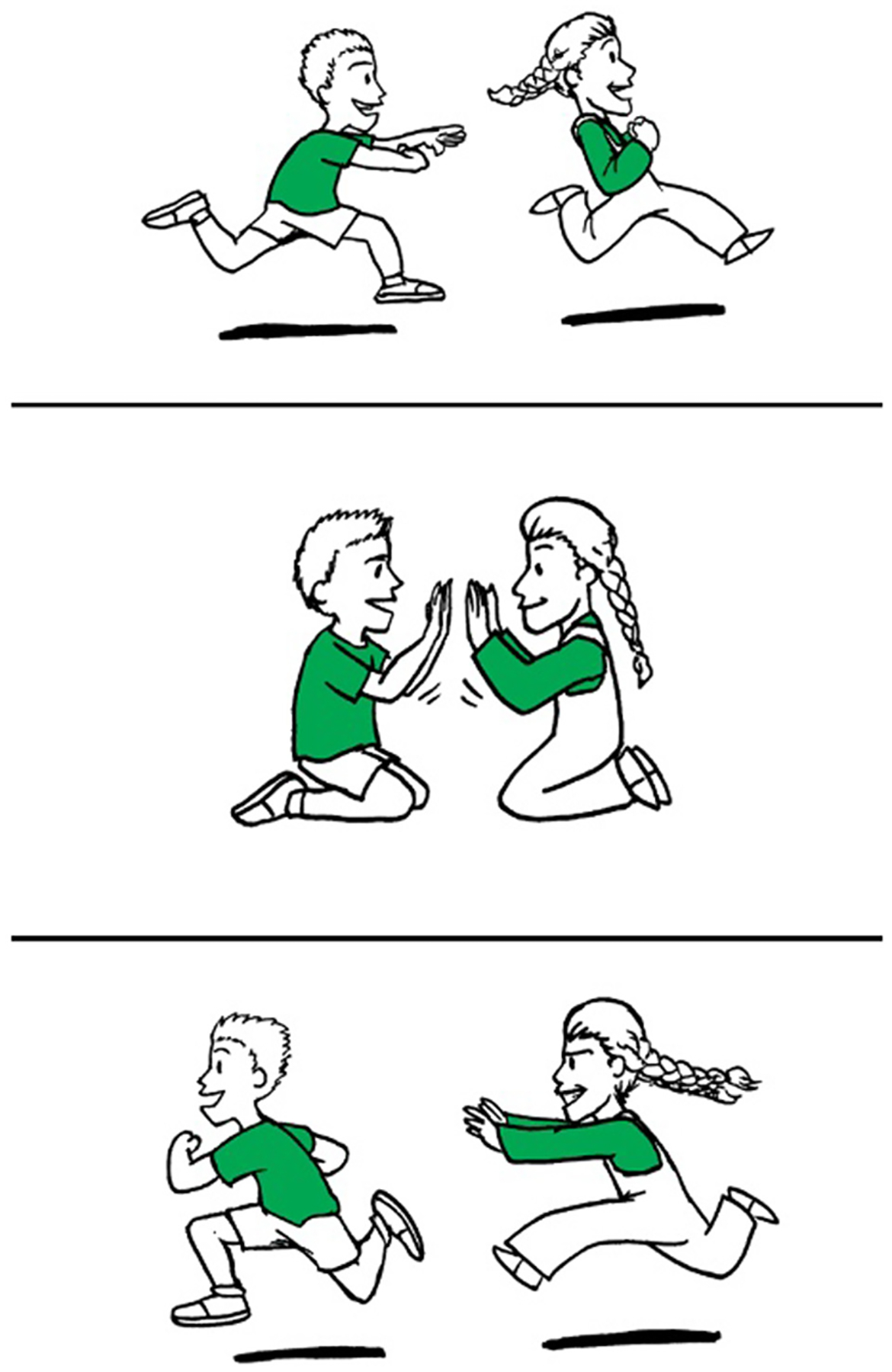
Sample item from the *SOAP Syntactic Battery of Sentence Comprehension* for Study 1. If the sentence accompanying this item is, “The boy chases the girl with the green shirt,” then the top picture is the Match, the middle picture is the Distractor, and the bottom picture is the Mismatch. The order of Match, Distractor, and Mismatch images was pseudorandomized.

For *E-SOAP* sentences, all descriptive phrases (e.g., “in the green shirt,” “wearing the blue pants”) were within a noun phrase either in the middle or at the end of the sentence, so that syntactic complexity was not impacted. Adjectives describing the characters varied in presence and type across sentences (e.g., “small” describes “boy” in 5a but not in 5b–d) to control word count and ensure that each sentence type (i.e., A, P, SR, OR) did not differ significantly in number of words (*M* = 10.3 words, *SD* = 0.56, range: 9–12 words; *F*[3, 36] = 2.59, *p* = 0.07, *η*^2^ = 0.18). Sentences were pseudorandomized within the test so that no more than two items of a particular syntactic structure were presented consecutively. Each sentence was accompanied by three simple line drawings involving two characters that were presented vertically (see Figure 2). One line-drawing corresponded to the correct answer (match condition) in which the agent and patient accurately engaged in the described action, another picture corresponded to an incorrect answer (mismatch condition) which involved the correct characters but a reversal of thematic roles so that the patient was depicted as the agent, and the third picture corresponded to a distractor answer which consisted of the relevant characters engaging in an unrelated action (distractor condition). Defining modifiers (such as color of hair, shirt, pants, etc.) were the same for each character per item to avoid these features serving as possible non-syntactic cues. The vertical ordering of the match, mismatch, and distractor images was pseudorandomized across test items.

For each trial on the *E-SOAP*, participants first listened to a pre-recorded narrator identify each character in the pictures to familiarize the participants with the characters and ensure that incorrect responses were not due to a lack of semantic knowledge regarding the characters (e.g., for Figure 2, the narrator would say “This is the doctor” and “This is the soldier” as each character was pointed to with an arrow across the three pictures). Next, participants heard the recorded sentence twice with 3,800 ms of silence between the first and second iteration of the sentence. Sentences and character introductions were pre-recorded by a native speaker of English using a typical conversational rate of speech in English [214 ± 6.73 words per minute (WPM); Yuan et al., 2006] to ensure participants could not use inadvertent intonation or rate of speech cues. Sentence recording length (in seconds; *M* = 2.75 s; *SD* = 0.27; range: 2.34–3.30 s) and rate of speech (WPM; *M* = 226.9 WPM; *SD* = 26.11; range: 164.4–276.5 WPM) did not significantly differ across sentence types (i.e., A, P, SR, OR; recording length: *F*[3, 36] = 0.81, *p* = 0.50, *η*^2^ = 0.06; rate of speech: *F*[3, 36] = 0.96, *p* = 0.43, *η*^2^ = 0.07).

All stimulus pictures, character introductions with arrow points, and audio recordings were programmed into PowerPoint and shown to participants on a laptop using headphones with volume comfortably adjusted. After hearing the sentence recording twice, participants were then asked to choose which picture correctly depicted the sentence they had just heard using a finger point that the experimenter recorded on a response form. Before starting the 40 experimental items, five practice items (involving canonical active and subject-relative constructions only) were administered, during which participants were given feedback regarding accuracy, and errors were reviewed to ensure comprehension of the instructions and the task. Each practice item was repeated until the participant reached 100% accuracy, demonstrating that they understood the task.

#### 2.2.3 Mandarin adaptation of the English SOAP syntactic battery of sentence comprehension

The *E-SOAP* was adapted into Mandarin (*M-SOAP*) by a research team that included trained linguists and native Mandarin speakers. Adapted sentences maintained mostly consistent structures within each syntactic category (i.e., subject-relative, object-relative, active, passive) as their English counterparts. Sentences 6a–d depict sample *M-SOAP* sentences. All *M-SOAP* sentences from Study 1 can be found in Appendix 2.


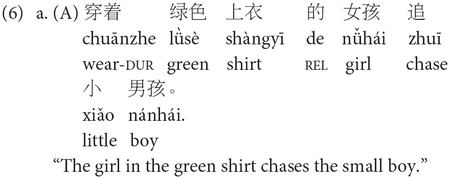



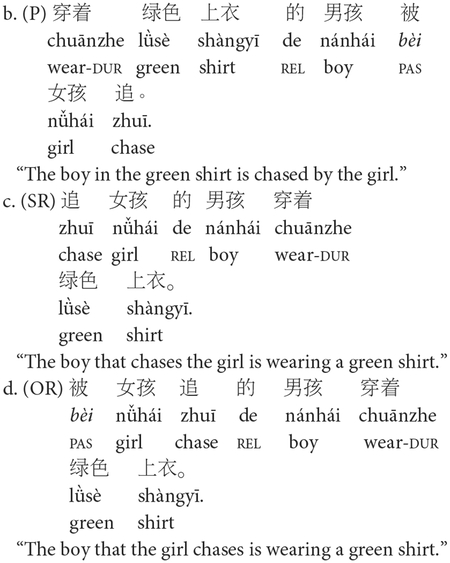


Contrary to the *E-SOAP* sentences, descriptive phrases (e.g., “in the green shirt,” “wearing the blue pants”) were either placed at the front of the sentence (in actives and passives) or at the end of the sentence (subject- and object-relatives). This was done because such descriptive phrases in Mandarin must come before the head noun they are describing (i.e., head-initial property of Mandarin). As such, we did not place such descriptors in positions where they would interrupt the flow of the sentence, potentially placing an extra burden on memory or increasing syntactic complexity. Instead, we opted to put them either at the beginning or end of the sentence, whichever was syntactically plausible for the given sentence construction. A sentence-initial passive marker (*bèi*) was added to the Mandarin object-relative constructions as it is strongly preferred by native speakers to improve linguistic acceptability, though it is not strictly required.

During the adaptation process, if an English word within a sentence did not have an equivalent Mandarin translation, the word was removed or substituted as appropriate (e.g., Mandarin does not have a concise translation equivalent of the English word “bedridden,” so this word was removed on the *M-SOAP*). After the team had adapted the *E-SOAP* sentences into Mandarin, a separate group of Mandarin-English bilingual speakers back-translated the adapted Mandarin sentences to English to see if the meaning was retained from the original *E-SOAP* sentences. Sentence length, measured by Chinese character count,^6^ differed significantly across the four sentence types (i.e., A, P, SR, OR; *M* = 13.2 characters/syllables, *SD* = 2.01, range: 10–17 characters/syllables; *F*[3, 36] = 5.12, *p* = 0.005, *η*^2^ = 0.30). These differences were driven by passives (non-canonical in Mandarin; *M* = 11.6 characters, *SD* = 2.22) being shorter than object-relatives (canonical in Mandarin; *M* = 14.6 characters, *SD* = 1.35; *p* = 0.003). All sentences and character introductions were recorded by a native Mandarin speaker around a typical conversation rate [247 ± 10.2 characters per minute (CPM); Yuan et al., 2006] where rate of speech did not significantly differ across sentence types (*M* = 273.6 CPM, *SD* = 22.8, range: 207.2–340.2 CPM; *F*[3, 36] = 1.14, *p* = 0.35, *η*^2^ = 0.09). As expected, sentence recording length (in seconds) was strongly correlated with sentence length measured via number of characters (*r*[38] = 0.87, *p* < 0.001). Thus, recording length also differed significantly across the four sentence types, reflecting differences in character count (*M* = 2.89 s, *SD* = 0.44, range: 2.16–3.77 s; *F*[3, 36] = 2.93, *p* = 0.047, *η*^2^ = 0.20) where passives (non-canonical in Mandarin; *M* = 2.64 s, *SD* = 0.44) were again significantly shorter than object-relatives (canonical in Mandarin; *M* = 3.17 s, *SD* = 0.31; *p* = 0.04). To account for potential effects of sentence length, this variable was included as a covariate in the analyses (described in Section 2.3.3). The same picture stimuli, vertical ordering of stimuli for each trial, and order of trials across the test were adopted from the *E-SOAP*. Identical procedures were followed when administering the *E-SOAP* and *M-SOAP*, and the two measures were always administered on separate days.

### 2.3 Analyses

#### 2.3.1 Reliability

Scoring was double-checked by a trained research assistant on 75% of the *E-* and *M-SOAP* assessments and on 100% of the *MoCA-BJ*. Overall point-to-point agreement between the primary examiner and research assistant was 100% for the *E-* and *M-SOAP* assessments and *MoCA-BJ*. Twenty-eight percent of verbal fluency data were scored a second time by a trained research assistant using audio recordings, where reliability was 96%.

#### 2.3.2 Language dominance score

A language dominance score was calculated by averaging across the *LEAP-Q* self-ratings for speaking, comprehension, and reading, as well as the verbal fluency score for groceries, as these were all correlated with one another, suggesting a shared underlying proficiency construct (all *r*’s ≥ 0.44, all *p*’s ≤ 0.07, for a similar approach to calculating language dominance, see Robinson Anthony and Blumenfeld, 2018). Verbal fluency for animals was not included due to reports of nonequivalence for this semantic category across English and Mandarin, given the salience of the Zodiac animals in Mandarin (Eng et al., 2019; Sung et al., 2025) that came out after Study 1’s design was conceived and data collection was carried out. Current exposure ratings across English and Mandarin were also not included in the overall language dominance score as these ratings did not correlate with the other included same-language metrics (−0.21 ≤ all *r*’s ≤ 0.22; 0.4 ≤ all *p*’s ≤ 0.9). Participants in Study 1 were all Mandarin dominant (*M* = 0.47, *SD* = 0.19, range: 0.12–0.79; the language dominance score can be interpreted on a scale from −1 to 1 where a score closer to −1 indicates an English-dominant participant, a score of 0 indicates a balanced bilingual, and a score closer to 1 means the participant is Mandarin-dominant). At the individual level, all participants reported higher proficiency in and exposure to Mandarin compared to English and demonstrated stronger (*n* = 17) or equal (*n* = 1) verbal fluency abilities in Mandarin compared to English.

#### 2.3.3 SOAP sentence analysis

We compared performance accuracy across sentences in each sentence pair (i.e., actives vs. passives and subject-relatives vs. object-relatives) and more broadly across all sentence types that follow a canonical vs. non-canonical word order. Recall that in English and Mandarin, canonical thematic patterns consist of the agent preceding the patient. In English, active and subject-relative constructions follow the canonical pattern, while passive and object-relative constructions exhibit non-canonical word order where the patient precedes the agent. On the other hand, in Mandarin, active and object-relative constructions are canonical while passive and subject-relative constructions are non-canonical. Statistical analysis of sentence comprehension accuracy was performed with R software (R Core Team, 2022) using the package *lme4* to compute mixed effects models (Bates et al., 2014) and the package *car* to compute analysis of deviance tables for the fixed effects of the mixed effects models (Fox and Weisberg, 2011). Sentence comprehension accuracy was analyzed altogether collapsing across both languages, Mandarin and English, and sentence types using logistic mixed effects models (Baayen et al., 2008) to test for fixed effects of Language (Mandarin and English), Canonicity (canonical and non-canonical), Presence of Relative Clause (subject-relatives and object-relatives vs. actives and passives), as well as interaction effects, while controlling for random effects of Participant and Item. The selected fixed effects allowed for investigation of canonicity more broadly, while also permitting further follow-up planned comparisons of performance across the specific sentence pairs of interest (i.e., actives and passives; subject- and object-relatives). We also included sentence length (measured in seconds) as a covariate in the model to account for differences in length across the Mandarin sentence types. To evaluate fixed effects, we report (a) raw regression coefficients (*β*), standard errors (*SE*), and Wald *Z* statistics from the model summary (coefficient-level tests); and (b) Wald chisquare (*χ*^2^) and associated *p*-values from the analysis of deviance tables (term-level tests). These approaches provide complementary information where coefficient-level tests indicate the size and direction of individual parameter estimates, whereas term-level tests evaluate the overall contribution and statistical significance of predictors. For follow-up planned comparisons to investigate significant interactions, we used the *emmeans* package to compute pairwise contrasts. We report log-odds estimates (*β*), *SE*s, Wald *Z* statistics, and false discovery rate (FDR)-corrected *p*-values, and we additionally present odds ratios, their 95% confidence intervals (CIs), and Cohen’s *d* effect sizes to facilitate interpretation of effect magnitude. R code for all analyses is available through the Open Science Framework (https://osf.io/h5g7w/?view_only=1432aed8289b44c4b7c04e4a51b4e254).

In addition to examining mean performance across sentences and languages, we also analyzed performance variability across participants, as this metric can reveal whether some sentence conditions elicit more consistent performance than others (Rutter et al., 2020). To look more closely at performer levels (e.g., higher vs. lower performers), we utilized Wilcoxon Signed Rank Testing, a nonparametric paired *t*-test, given the smaller sample size after dividing our sample into higher and lower performers. For Wilcoxon Signed Rank Testing, we report *Z* values, two-tailed *p*-values, and effect sizes (*d*).

Regarding our hypotheses and expected results, if *structural-based* accounts best reflect performance cross-linguistically, we would expect to see no effect of Canonicity because such an effect would indicate different patterns of performance for canonical and non-canonical sentences across languages, which constitute different sentence types across English and Mandarin. If *top-down canonicity* accounts best fit the data cross-linguistically, then we would expect to observe an effect of Canonicity and possibly a Language × Canonicity interaction effect if the patterning of performance across canonical and non-canonical sentences differs in any way across English and Mandarin. Any obtained effects of Language may be due to differences in proficiency across English and Mandarin in our bilingual participants. Any effects involving the Relative Clause term will not be interpreted, as our primary research questions were aimed at investigating performance between sentences in pre-determined pairings (i.e., actives and passives; subject- and object-relatives) rather than exploring the difference in performance across sentences with and without relative clauses. However, this term was included to investigate canonicity while also conducting *post-hoc* analyses between sentences in the designated pairs.

### 2.4 Results

#### 2.4.1 Group results

**FIGURE 3:**
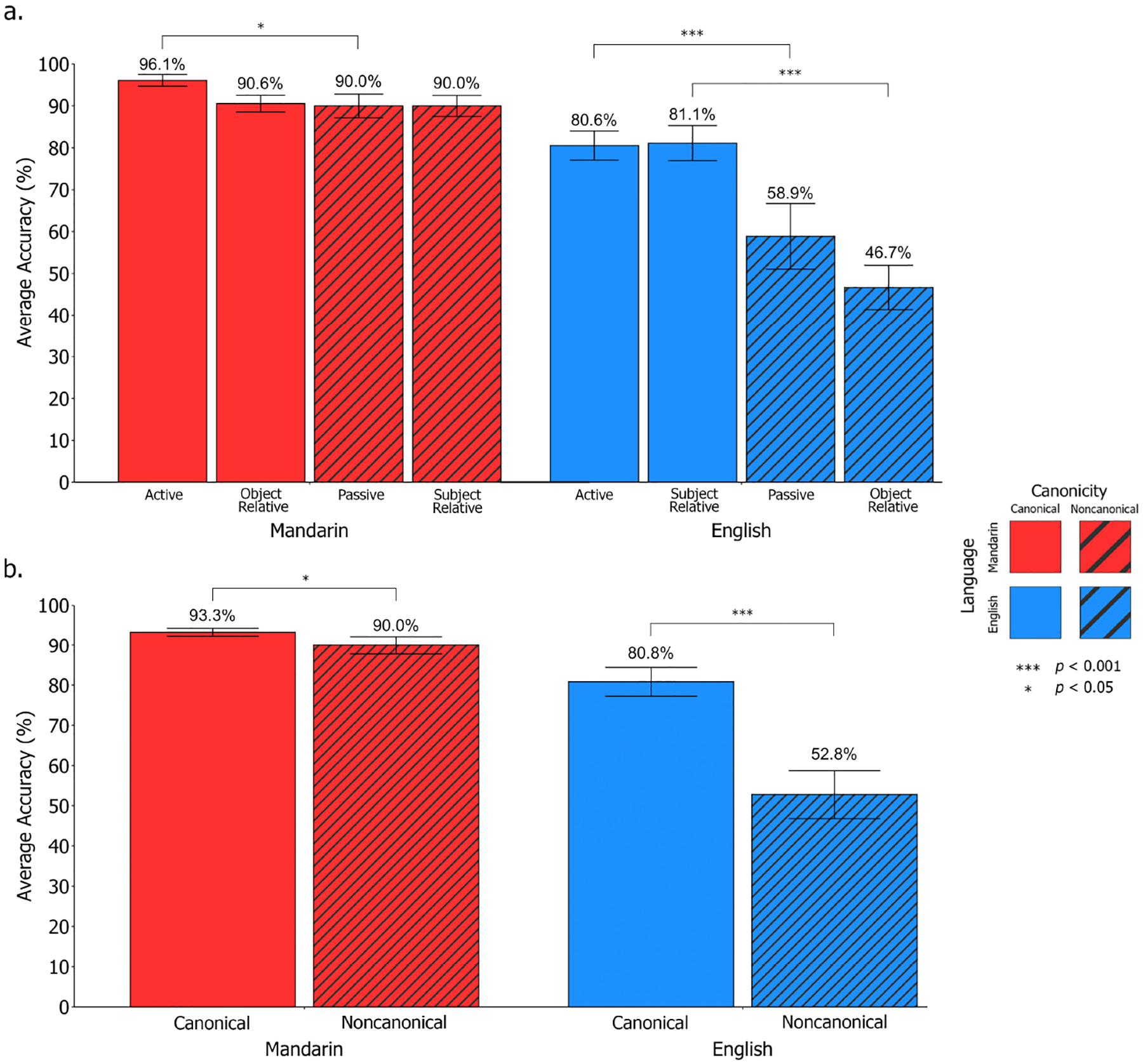
Study 1 percent accuracy for all sentence types across Mandarin and English **(a)**. Study 1 percent accuracy on canonical and non-canonical sentences across Mandarin and English **(b)**. ***p < 0.001; *p < 0.05.

Through logistic mixed effects modeling, we obtained the following relevant significant^7^ main and interaction effects: (1) Language; (2) Canonicity; and (3) Language × Canonicity. Regarding the effect of Language, participants performed significantly better in Mandarin compared to English (*χ*^2^[1,18] = 122.95, *p* < 0.001; *β*_*raw*_ = 1.98, *SE* = 0.44, Wald *Z* = 4.50; *M_Mandarin_* = 91.7%, *M_English_* = 66.8%). For the effect of Canonicity, participants performed significantly better on canonical structures compared to non-canonical structures (*χ*^2^[1,18] = 48.44, *p* < 0.001; *β*_*raw*_ = 1.27, *SE* = 0.30, Wald *Z* = 4.19; *M*_*canonical*_ = 87.1%, *M*_*non*−*canonical*_ = 71.4%). When examining the Language × Canonicity interaction effect, participants demonstrated an improvement in performance for English canonical sentences compared to non-canonical sentences, where this pattern was not as pronounced in Mandarin (*χ*^2^[1,18] = 4.70, *p* = 0.03); however, the raw coefficient estimate (*β_raw_* = 0.06, *SE* = 0.53, Wald *Z* = 0.12)^8^ indicates a small effect size (see Figures 3a, b).

Planned follow-up comparisons were conducted to break down the significant Language × Canonicity interaction effect, indicating that in English, participants performed significantly better on canonical compared to non-canonical sentences (*β* = 1.55, *SE* = 0.22, *z* = 7.14, *p* < 0.0001, Cohen’s *d* = 0.86, odds ratio = 4.71, 95% CI: [3.08, 7.21]). Performance was significantly above chance for English canonical sentences (*t*[17] = 8.62, *p* < 0.001) and at chance-level for English non-canonical sentences (*t*[17] = 0.47, *p* = 0.65; chance level was set at 50% given that two of three picture options in each *SOAP* trial were relevant to the trial sentence). In Mandarin, the difference between participants’ performance on canonical and non-canonical sentences was smaller but still significant (*β* = 0.70, SE = 0.32, *z* = 2.15, *p* = 0.03, Cohen’s *d* = 0.38, odds ratio = 2.01, 95% CI: [1.06, 3.78]) where participants performed above chance-level across both Mandarin canonical (*t*[17] = 43.8, *p* < 0.001) and non-canonical sentences (*t*[17] = 18.1, *p* < 0.001). Further planned follow-up comparisons were conducted to investigate performance between specific sentence pairs of interest (i.e., actives vs. passives; subject-relatives vs. object-relatives). In English, participants performed significantly better on actives compared to passives (*β* = 1.27, *SE* = 0.30, *z* = 4.19, *p* = 0.0001, Cohen’s *d* = 0.70, odds ratio = 3.56, 95% CI: [1.96, 6.45]) and on subject-relatives compared to object-relatives (*β* = 1.83, *SE* = 0.30, *z* = 6.03, *p* < 0.0001, Cohen’s *d* = 1.01, odds ratio = 6.25, 95% CI: [3.45, 11.32]). In Mandarin, participants performed significantly better on actives compared to passives (*β* = 1.21, *SE* = 0.50, *z* = 2.43, *p* = 0.02, Cohen’s *d* = 0.67, odds ratio = 3.35, 95% CI: [1.26, 8.88]). No such difference was observed between subject- and object-relatives in Mandarin (*β* = 0.18, *SE* = 0.40, *z* = 0.46, *p* = 0.70, Cohen’s *d* = 0.10, odds ratio = 1.20, 95% CI: [0.55, 2.64]).

#### 2.4.2 Variability and individual level performance in Mandarin

**FIGURE 4:**
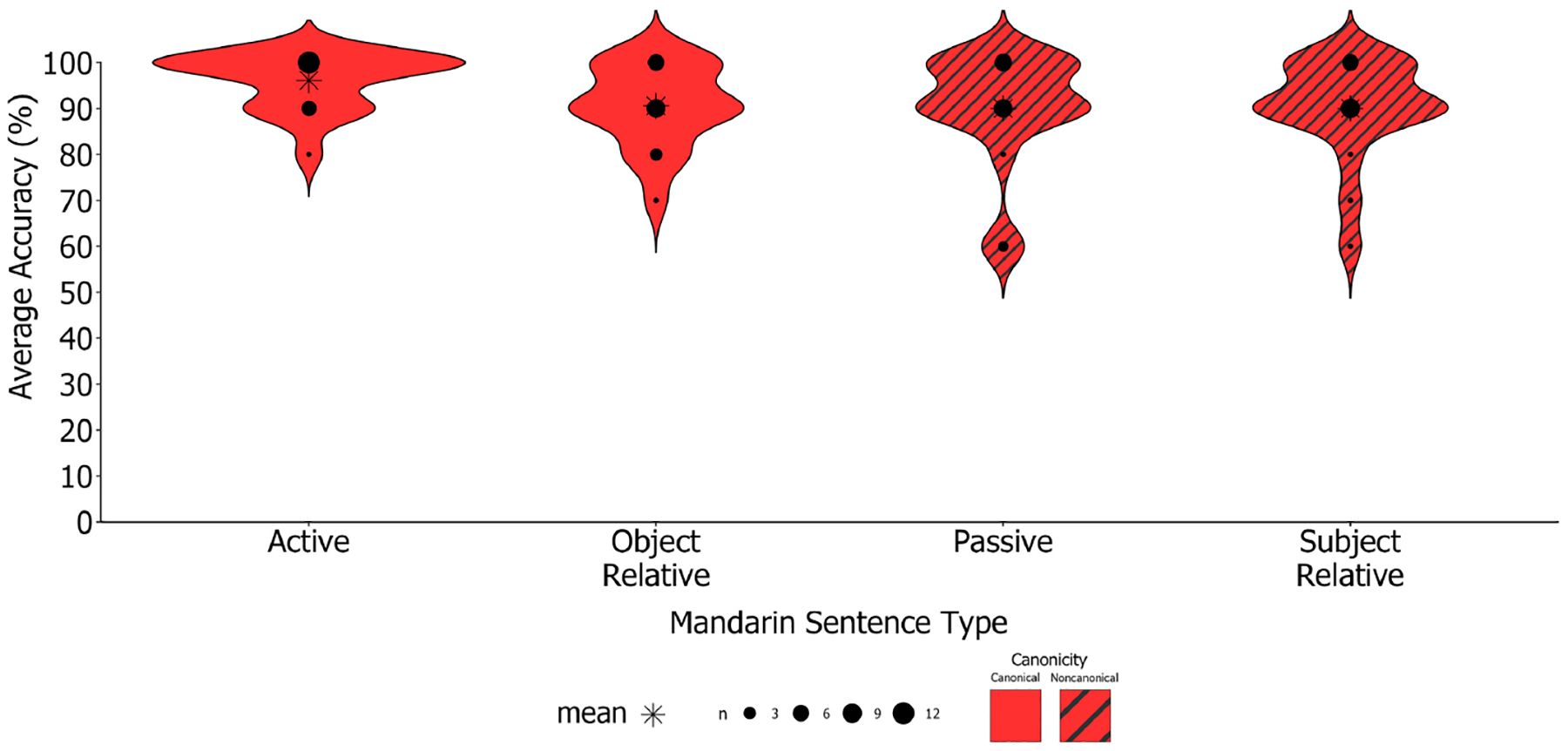
Study 1 distribution of performance across sentences in Mandarin. Non-canonical sentences (passives and subject-relatives) display a wider performance distribution, indicating increased variability in performance. *Indicates the mean accuracy for each sentence type in Mandarin while the size of the points indicates the number of participants performing at that level of accuracy.

Despite high overall performance on sentences in participants’ dominant and early learned language of Mandarin, participants performed more variably on Mandarin passives and subject-relatives (both non-canonical sentences; SD_*non*−*canonical*_ = 9.20) vs. canonical sentences (i.e., actives and object-relatives; SD_*canonical*_ = 4.10; see Figure 4 for distribution of performance).

**FIGURE 5:**
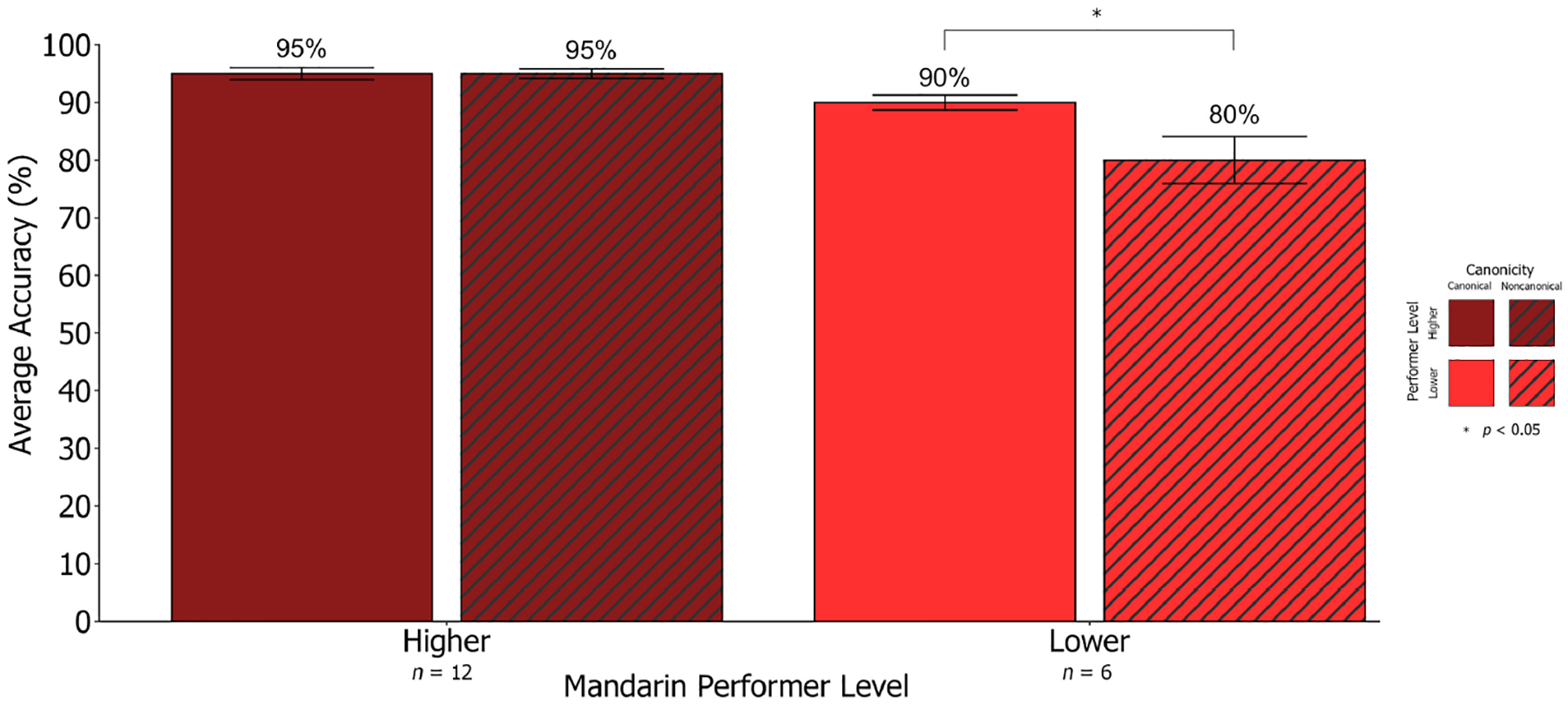
Study 1 average accuracy for Mandarin canonical and non-canonical sentences across performer levels. Higher performers = above average (91.7%) performance across all Mandarin sentences (*n* = 12); lower performers = below average performance across all Mandarin sentences (*n* = 6); *p < 0.05.

Given the increased variability in performance for non-canonical sentences in Mandarin, we conducted further analyses by splitting participants into higher (i.e., near ceiling-level) and lower performers based on their performance across all the *M-SOAP* sentences. Participants were classified as higher performers (*n* = 12) if they performed above the overall group average on the *M-SOAP* (91.7%), with all others classified as lower performers (*n* = 6). Using Wilcoxon Signed Rank Testing to compare performance between performer groups, we found that higher and lower performers demonstrated differing patterns of performance across Mandarin canonical and non-canonical sentences. Higher performers performed at the same level of accuracy on average (95%) across Mandarin canonical and non-canonical sentences. Conversely, lower performers displayed significantly lower accuracy on Mandarin non-canonical (80%) compared to canonical sentences (90%; *Z* = −2.05, *p* = 0.04, *d* = 0.84, *n* = 6; see Figure 5). Lower performers’ worse non-canonical performance was driven by lower accuracy for passive sentences (78.3%) compared to active sentences (95.0%; *Z* = −2.47, *p* = 0.01, *d* = 1.01, *n* = 6). Performance did not significantly differ across subject- (81.7%) and object-relative (85.0%) sentences for lower performers (*Z* = 0.47, *p* = 0.64, *d* = 0.19, *n* = 6). Thus, overall performance on the *M-SOAP* appears to be constrained by canonicity.

### 2.5 Interim discussion

From Study 1, we can see that canonicity effects were apparent in English, which was a lower-proficient and later-learned language, and in Mandarin, a native and earlier-learned language. English results confirmed findings from prior literature of a performance advantage for English active and subject-relative constructions (both exhibiting canonical word order, e.g., Love and Oster, 2002). The current findings add to the previous literature in showing that this canonicity effect is maintained when English is a relatively low-proficient and late-acquired language. Additionally, Mandarin findings replicated prior work depicting high performance overall with a decrease and added variability for non-canonical sentences (especially for passive sentences in below-average performers) when participants are tested in their native language (e.g., Love and Oster, 2002). Moreover, patterns observed in Study 1 suggest that canonicity effects converge somewhat across languages, with the passive-active contrast present in both languages, but that there is clear divergence regarding the subject-relative advantage that is present in English but not in Mandarin. Thus, Study 1 findings do not clearly support structural-based accounts, which predict the same pattern across English and Mandarin: higher accuracy for subject-relatives than object-relatives (not observed in Mandarin), and for active sentences than passives (observed across English and Mandarin). The results also do not entirely align with canonicity-based accounts. These accounts predict higher accuracy for object-relatives over subject-relatives in Mandarin (a pattern not observed), higher accuracy for subject-relatives over object-relatives in English (a pattern that was observed), and higher accuracy for actives than passives across both languages (a pattern that was observed). In Study 2, we attempt to resolve this ambiguity.

From Study 1 alone, it is unclear to what extent observed patterns are driven by language profiles, age, or stimulus characteristics. Obtained differences in sentence comprehension patterns across English and Mandarin are likely due to lower English proficiency. Further, it is unclear whether the lack of distinct performance across subject- and object-relatives in Mandarin was due to high Mandarin proficiency levels or other stimulus-related factors. Given the variability in object-relative performance in Study 1, it is possible that the sentence-initial *bèi* passive marker influenced comprehension and masked a subject-relative/object-relative performance difference. Although this marker increases linguistic acceptability for native speakers, it also adds an extra word, making it harder to directly compare subject-relatives and object-relatives in Mandarin. This may be especially relevant since object-relative constructions without the sentence-initial *bèi* marker are more commonly examined in the Mandarin sentence processing literature (e.g., Chen et al., 2008; Gibson and Wu, 2013; Hsiao and Gibson, 2003; Lin and Bever, 2006; Lin and Garnsey, 2011; Packard et al., 2011; Qiao et al., 2012; Sun et al., 2016; Sung et al., 2016; Vasishth et al., 2013; Xu et al., 2019; Yang et al., 2010; Yang and Perfetti, 2006).

## 3 Study 2: sentence processing in Mandarin–English bilinguals across language dominance and adult age ranges

We conducted a follow-up study, Study 2, to replicate and extend sentence comprehension findings while addressing the potential shortcomings in Study 1. We expanded the Study 1 *M-SOAP* sentences for Study 2 to include an additional set of 10 object-relatives without the sentence-initial *bèi* passive marker. These 10 newly included object-relative sentences without the sentence-initial *bèi* marker allow for a more direct comparison between object-relative and subject-relative constructions in Mandarin, as the two constructions now have the same number and types of words, but with differing word order. In addition, based on careful item analysis of Study 1 results, and in consultation with native Mandarin speakers, we further revised the Mandarin sentences to ensure linguistic acceptability and cultural alignment (see Section 3.2.3). Moreover, to better understand if language proficiency was modulating effects, we recruited a sample with a broader range of linguistic profiles for Study 2. Participants in Study 2 were bilinguals across the lifespan (*M*_age_ = 42.4; *SD* = 20.8) who ranged from Mandarin dominant to English dominant and balanced bilingualism with varying proficiency and exposure levels across Mandarin and English (see Sections 3.1 and 3.3.2, Table 3). Finally, as all participants in Study 1 were older adults (*M*_age_ = 76.8; *SD* = 4.08), we recruited participants with a wider age range to rule out that patterns were due to potential age-related factors.

### 3.1 Participants

Thirty-six Mandarin-English bilingual adults were recruited from Southern and Northern California via announcements and flyers. Three participants reported a history of a head injury during childhood involving loss of consciousness but were not excluded as their cognitive performance on the *MoCA* was comparable to other participants and within 1.5 standard deviations of the language-specific cut-off used to differentiate mild cognitive impairment from typical functioning (Wei et al., 2024; Nasreddine et al., 2005). One participant reported a history of attention-deficit hyperactivity disorder diagnosis, but outcomes of all analyses remained the same when this participant was excluded from analyses, so the participant remained in the current cohort. One participant did not pass the hearing screening and was excluded, thus resulting in a total of 35 participants that were included in Study 2 (*M*_*age*_ = 42.43, *SD* = 20.76, range: 18–85, 27 female, eight male). Study 2 followed the same recommendations of San Diego State University’s Institutional Review Board from Study 1, and similar vision and hearing screeners were utilized that participants had to pass to participate. Twenty-four participants reported immigrating to the United States, with a mean age of immigration of 23.71 years (*SD* = 11.10, range: 8–51 years). The *LEAP-Q* (Marian et al., 2007; Blumenfeld et al., 2017) and *Multilingual Naming Test-Sprint Version* (*MINT-Sprint*; Garcia and Gollan, 2022) were utilized to characterize participants’ linguistic profiles. Participants demonstrated a range of language dominance scores that were composed of their self-reported and objective proficiencies as well as exposure to each language (*M* = 0.02; *SD* = 0.32; on a scale from −1 to 1 where 0 indicates balanced bilingualism; see Section 3.3.2). Participants reported Mandarin as their L1 (*n* = 18), L2 (*n* = 10), or L3 (*n* = 7), and English as their L1 (*n* = 3), L2 (*n* = 7), L3 (*n* = 17), L4 (*n* = 7), or L5 (*n* = 1). Chinese dialects were considered their own language when assigning order of language acquisition, and individuals who reported Mandarin as their L2 or L3 had other Chinese dialects as languages learned before Mandarin. Other languages that were acquired by participants included Cantonese, Taiwanese (all *n* = 11); Japanese (*n* = 9); Spanish (*n* = 5); French (*n* = 4); Burmese (*n* = 3), Shanghainese, Fujianhua (all *n* = 3); Sichuanese, Hakka (all *n* = 2); Korean, Vietnamese, Portuguese, Qingdaohua, Shanxihua, Shanbeihua, Hubeihua, Fuzhouhua, Longduhua, and Hangzhouhua (all *n* = 1). Table 3 summarizes participants’ linguistic and cognitive profiles. All participants were offered compensation for their participation.

### 3.2 Procedure, materials, and experimental design

Participants completed the following tasks for Study 2: (1) an oral interview version of the *LEAP-Q* (Marian et al., 2007; Blumenfeld et al., 2017); (2) the English and Mandarin *MINT-Sprint* (Garcia and Gollan, 2022); (3) the *MoCA* in their preferred language (Nasreddine et al., 2005; Zheng et al., 2012); and (4) the *E-SOAP* (Love and Oster, 2002) and the revised *M-SOAP* (described in Section 3.2.3). All task instructions were pre-recorded in Mandarin and English by native speakers. Tasks were administered individually by trained Mandarin-English bilingual researchers in quiet testing rooms at San Diego State University or other preferred community-based locations (e.g., a local library near the participant’s home) upon participant request. Sessions were generally conducted such that all tasks were completed in the participants’ preferred language first, followed by tasks in the less preferred language to allow participants the opportunity to understand the format and expectations of tasks with minimal clarification necessary due to language proficiency. Data were recorded manually and automatically through button press for the *SOAP* tasks, as well as audio and video recorded, with reliability checks in place (described in Section 3.3.1).

#### 3.2.1 Language history, proficiency, and cognitive background measures

The oral interview version of the *LEAP-Q* (Blumenfeld et al., 2017; Marian et al., 2007) was administered either in English, Mandarin, or a combination of the two languages via a bilingual format.

The *MINT-Sprint* (Garcia and Gollan, 2022) is a rapid naming test, based on a subset of items from the longer *Multilingual Naming Test* (*MINT*) that was specifically developed to assess bilingual language proficiency across languages, including English and Mandarin versions (Gollan et al., 2012). The *MINT-Sprint* consists of 80 colored pictures ordered by difficulty that are presented simultaneously in an eight-by-ten grid on a laptop screen. Participants had 3 min to name as many pictures as they could, as quickly as possible, starting at the top left corner and proceeding across each row. The 3-min cutoff was not imposed but only intended to give participants a sense of time pressure. After participants finished, they were prompted to take a second pass through the pictures they had skipped or named incorrectly in the first pass. An overall *MINT-Sprint* raw score was calculated by summing up all correctly named items from the first and second pass naming attempts. A gap of at least 30 min was imposed between administration of each language’s *MINT-Sprint* during which participants completed other tasks.

Based on participants’ language preference and abilities gathered from the *LEAP-Q*, either the English *MoCA* (Nasreddine et al., 2005) or the *Chinese-Language Los Angeles Version of the MoCA* (*MoCA-ChLA*; Zheng et al., 2012) or a mixture of the two assessments was administered to characterize cognitive abilities (Brice et al., 2014). The *MoCA-ChLA* was selected for Study 2 as it was specifically normed on a population of Chinese speakers in Los Angeles, California and all participants for Study 2 resided in California. All included participants performed within 1.5 standard deviations of typical language-specific cut-off scores on the *MoCA* or *MoCA-ChLA* for their age range (*M* = 28.2, *SD* = 1.7, range: 24–30; Nasreddine et al., 2005; Hong et al., 2022; Wei et al., 2024).

#### 3.2.2 SOAP syntactic battery of sentence comprehension

For Study 2, the *E-* and *M-SOAP* were programmed and administered via SuperLab Version 4.5 on a laptop computer, allowing for accuracy data to be collected automatically (see Figure 2 for an example). Administration procedures were similar to Study 1 (see Section 2.2.2) except that participants were instructed to press keys on the number pad portion of the keyboard to indicate their response (“9” for top picture, “5” for middle picture, and “1” for bottom picture). Participants were told to wait until the end of the first sentence before responding and were allowed to listen to the sentence a second time if needed, which automatically played 3,800 ms after the first sentence if the participant did not respond. If participants needed to hear the second sentence repetition, they were allowed to respond at any point during or after the second sentence. No time limit was imposed on responses. A blank white screen was shown for 1,000 ms between each test item. Participants were given breaks and shown their progress in the task after every 10 items. Five practice items were administered at the start of the task where participants were given audio-recorded and typed feedback regarding their accuracy and speed, such as being told if they responded too quickly within the first sentence iteration. Participants completed each practice item until they achieved 100% accuracy before proceeding to the test items.

#### 3.2.3 SOAP syntactic battery of sentence comprehension changes across studies 1 and 2

The *E-SOAP* test items were further pseudorandomized for Study 2 to ensure that no more than two items of a particular syntactic structure (i.e., A, P, SR, OR) were presented consecutively and that no more than two non-canonical or canonical structures appeared consecutively. Test items involving the characters of an Indian and cowboy were replaced with the characters of a gardener and painter, respectively, on both the *E-* and *M-SOAP* due to cultural incongruence of the original items that was revealed through item analyses of Study 1 data.

The *M-SOAP* was further adapted from Study 1 to Study 2 to improve linguistic acceptability. Specifically, two practice items from Study 1 were subject-relative sentences that, in Mandarin, follow a non-canonical structure where all practice items were intended to be canonical in structure. Thus, these two items were adjusted from non-canonical subject-relative structures to canonical object-relative constructions on the *M-SOAP*. Aspect markers were added where appropriate to ensure verbs followed all correct aspects as reflected by the picture stimuli (e.g., Study 1: 追/*zhuī*/“chase”; Study 2: 追 着/*zhuīzhe*/“chases [durative]”). Specific vocabulary choices in the target sentences were adjusted to better suit the semantic context of the picture stimuli (e.g., Study 1: 逮 捕/*dàibǔ*/“arrest” changed to Study 2: 抓 到/*zhuādào*/“catch” or “capture”). The linguistic structure of specific noun phrase descriptors was adjusted to enhance acceptability of these descriptive structures (e.g., Study 1: 金 发 的… /*jīnfǎ de…* /“blonde-haired… ”; Study 2: 有 着 一 头 金 发的… /*yŏuzhe yītóu jīnfă de…* /“… has [durative] [one-measure word] blonde hair”). To improve linguistic acceptability, 那个 *nàge* “that” was added before the second actor across all sentences on the *M-SOAP* as Mandarin does not have a translation equivalent of “the.” Three sentences with unintended intervening descriptors from Study 1 were modified to remove the intervening descriptor such that all descriptor phrases were at the beginning (actives and passives) or end of the sentence (subject and object-relatives) as was originally intended. Finally, while object-relative sentences in Study 1 were developed to start with the passive marker *bèi*, which indicates that the noun immediately following it will be the object of the sentence, this marker has not been included in previous literature comparing subject and object-relative sentences and thus a new object-relative construction without this sentence-initial *bèi* marker was included in Study 2 that was otherwise identical apart from the absence of the *bèi* marker. With the addition of 10 object-relative sentences without the *bèi* sentence-initial marker, the revised *M-SOAP* had a total of 50 sentences. All adjusted *M-SOAP* sentences used in Study 2 can be found in Appendix 3. Sentence length, measured in number of Chinese characters, differed significantly across the five sentence types (i.e., A, P, SR, OR, *bèi*-OR; *M* = 15.8 characters, *SD* = 0.70, range: 15–18 characters; *F*[4, 45] = 4.73, *p* = 0.003, *η*^2^ = 0.30). These differences were driven by two factors: (1) actives (canonical in Mandarin; *M* = 15.4 characters; *SD* = 0.52) were shorter than object-relative constructions with the sentence-initial *bèi* marker (*bèi*-OR; canonical in Mandarin; *M* = 16.4 characters; *SD* = 0.70; *p* = 0.004); and (2) *bèi*-OR constructions (*M* = 16.4, *SD* = 0.70) were longer than object-relative constructions without the *bèi* marker (OR; *M* = 15.4, *SD* = 0.70; *p* = 0.004), as expected. All sentences and character introductions were recorded by a native Mandarin speaker around a typical conversation rate [247 ± 10.2 characters per minute (CPM), Yuan et al., 2006], and rate of speech did not differ significantly across sentence types (*M* = 255.7 CPM, *SD* = 11.1, range: 233.4–292.0 CPM; *F*[4, 45] = 0.6, *p* = 0.67, *η*^2^ = 0.05). Sentence recording length (in seconds) was strongly correlated with sentence length in characters (*r*[48] = 0.71, *p* < 0.001) and also differed significantly across sentence types (*M* = 3.71 s, *SD* = 0.22, range: 3.29–4.28 s; *F*[4, 45] = 2.62, *p* = 0.048, *η*^2^ = 0.19). However, no follow-up pairwise comparisons indicated that any specific sentence type pairing accounted for this overall effect. Sentence length was included as a covariate in subsequent analyses, consistent with Study 1 (see Section 2.3.3).

### 3.3 Analysis

#### 3.3.1 Reliability

All *MINT-Sprint* and *MoCA* data were scored a second time by a trained research assistant using audio and video recordings. Reliability between primary and secondary scorers was calculated for 97% of the English *MINT-Sprint*, 100% of the Mandarin *MINT-Sprint*, and 100% of the *MoCA* tasks and were 99.8%, 97.9%, and 98.4%, respectively.

#### 3.3.2 Language dominance score

A language dominance score was calculated as described in Study 1 (see Section 2.3.2) and in Robinson Anthony and Blumenfeld (2018) by averaging across *LEAP-Q* self-ratings for speaking, comprehension, and reading, current exposure,^9^ as well as *MINT-Sprint* scores, as these variables were all correlated with each other warranting their combination (all *r*’s ≥ 0.47, all *p*’s ≤ 0.005). Participants demonstrated a range of language dominance scores with some being Mandarin-dominant, English-dominant, or balanced bilinguals (*M* = 0.02, *SD* = 0.32, range: −0.6 to 0.46). On average, however, Study 2 participants were more balanced bilinguals compared to the Mandarin-dominant participants from Study 1 (*M* = 0.47, *SD* = 0.19, range: 0.12–0.79). Moreover, on average, Study 2 participants performed similarly on the *MINT-Sprint* across languages (Mandarin: *M* = 74.8%, *SD* = 16.9; English: *M* = 76.6%, *SD* = 15.1; *p* = 0.55). They also reported on average roughly equal exposure across Mandarin (*M* = 46.7%, *SD* = 28.7) and English (*M* = 47.6%, *SD* = 29.5; *p* = 0.93). Subjectively, compared to Study 1, Study 2 participants reported relatively comparable proficiency in Mandarin (*M* = 8.5, *SD* = 1.9; out of 10) and English (*M* = 7.7, *SD* = 1.87; out of 10). However, this difference was still statistically significant (*p* = 0.04), with participants rating themselves lower in English. This difference in subjective self-ratings of proficiency may not reflect actual ability, as *MINT-Sprint* scores did not significantly differ across the two languages and may instead reflect cultural humility in self-ratings of language proficiency (Tomoschuk et al., 2019). All other analyses for Study 2 closely followed Study 1 (see Section 2.3.3).

### 3.4 Results

#### 3.4.1 Group accuracy results

**FIGURE 6:**
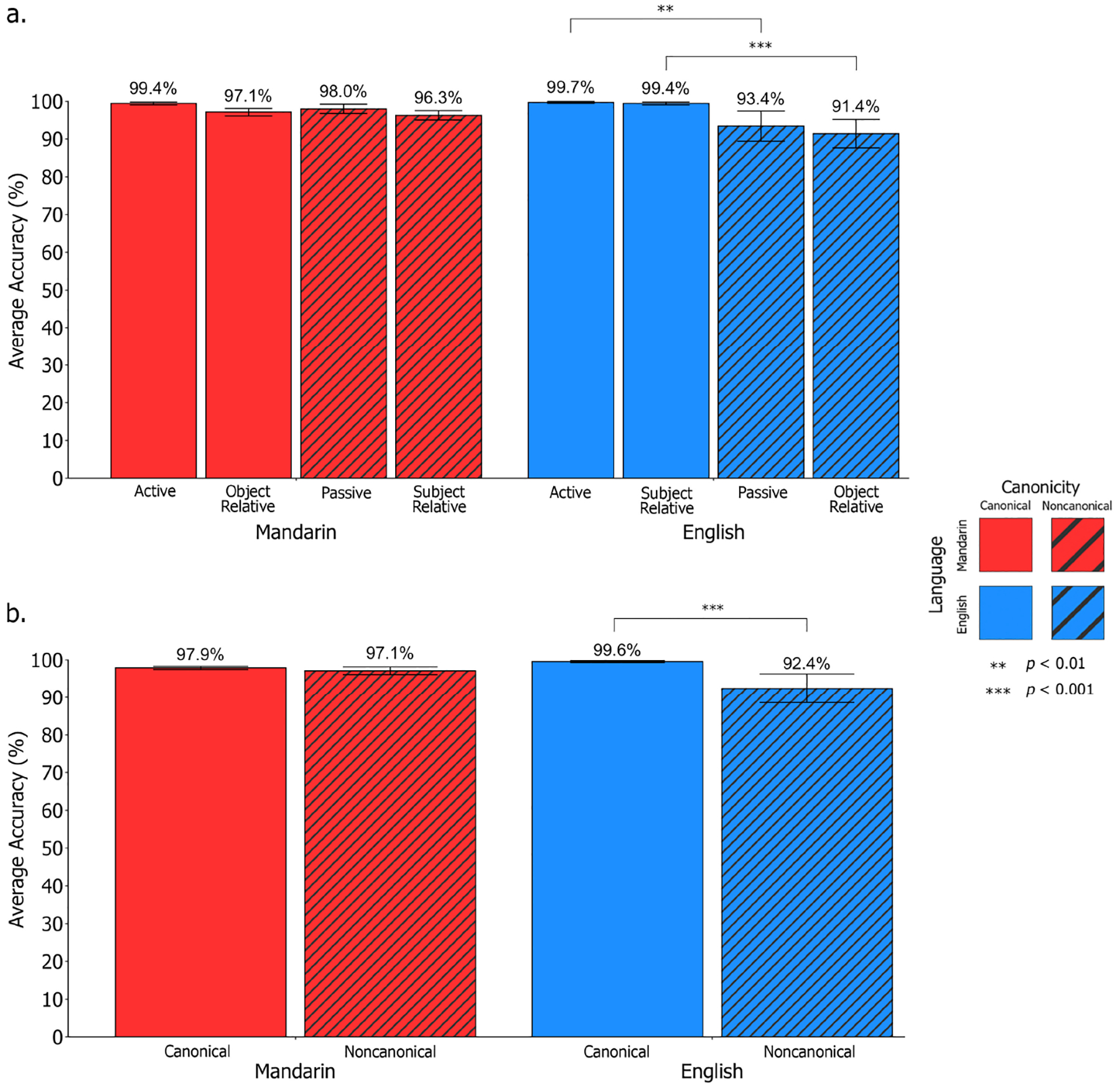
Study 2 percent accuracy for all sentence types across Mandarin and English **(a)**. Study 2 percent accuracy on canonical and non-canonical sentences across Mandarin and English **(b)**. **p < 0.01; ***p < 0.001.

For all statistical models, performance on object-relative constructions in Mandarin was collapsed across those with and without the sentence initial *bèi* marker given that performance did not significantly differ (*t*[34] = −0.39, *p* = 0.37; *M*_*b*è*i*−*OR*_ = 96.6%, *SD*_*b*è*i*−*OR*_ = 7.25*; M*_*OR*_ = 97.7%, *SD*_*OR*_ = 4.26). Using the same logistic mixed effects models from Study 1, we obtained the following relevant significant or near-significant^10^ main and interaction effects: (1) Language; (2) Canonicity; and (3) Language × Canonicity. All obtained effects replicate and confirm Study 1 findings. Regarding the effect of Language, although participants performed at a near-ceiling level across both languages, a near-significant difference between languages emerged where performance in Mandarin was marginally stronger (*χ*^2^[1,35] = 3.76, *p* = 0.053; *M_Mandarin_* = 97.6%, *M_English_* = 96.0%), however the raw coefficient estimate (*β*_*raw*_ = 0.36, *SE* = 1.31, Wald *Z* = 0.28) indicates a small effect size. For Canonicity, participants performed significantly better on canonical compared to non-canonical structures (*χ*^2^[1,35] = 15.3, *p* < 0.0001; *β*_*raw*_ = 3.52, *SE* = 1.04, Wald *Z* = 3.39; *M*_*Canonical*_ = 98.6%, *M*_*non*−*canonical*_ = 94.8%). When examining the Language × Canonicity interaction, participants demonstrated an improvement in performance for English canonical sentences compared to non-canonical sentences where this pattern was not as pronounced in Mandarin due to overall high performance in Mandarin (*χ*^2^[1,35] = 13.6, *p* = 0.0002, *β*_*raw*_ = 2.23, *SE* = 1.33, Wald *Z* = 1.68; see Figures 6a, b).

In both Mandarin and English, follow-up paired *t*-tests revealed that participants performed significantly above chance-level across canonical and non-canonical sentence structures (*t*_*Mandarin canonical*_[34] = 110.3, *p* < 0.001; *t*_*Mandarin non*−*canonical*_[34] = 45.7, *p* < 0.001; *t*_*English canonical*_[34] = 206.5, *p* < 0.001; *t*_*English non*−*canonical*_[34] = 11.0, *p* < 0.001). Planned follow-up comparisons were conducted to break down the significant Language × Canonicity interaction effect, indicating that in English, participants performed significantly better on canonical compared to non-canonical sentences (*β* = 3.33, *SE* = 0.64, *z* = 5.17, *p* < 0.0001, Cohen’s *d* = 1.84, odds ratio = 28.0, 95% CI: [7.91, 98.8]). In Mandarin, the difference between participants’ performance on canonical and non-canonical sentences was smaller and not statistically significant (*β* = 0.82, SE = 0.45, *z* = 1.79, *p* = 0.09, Cohen’s *d* = 0.45, odds ratio = 2.26, 95% CI: [0.93, 5.5]). Further planned follow-up comparisons were conducted to investigate performance between specific sentence pairs of interest (i.e., actives vs. passives; subject-relatives vs. object-relatives). In English, participants performed significantly better on actives compared to passives (*β* = 3.52, *SE* = 1.04, *z* = 3.39, *p* = 0.003, Cohen’s *d* = 1.94, odds ratio = 33.8, 95% CI: [4.42, 259]) and on subject-relatives compared to object-relatives (*β* = 3.14, *SE* = 0.75, *z* = 4.17, *p* = 0.0008, Cohen’s *d* = 1.73, odds ratio = 23.1, 95% CI: [5.28, 101]). In Mandarin, given the near-ceiling performance, no such differences were observed between actives and passives (*β* = 1.29, *SE* = 0.82, *z* = 1.57, *p* = 0.17, Cohen’s *d* = 0.71, odds ratio = 3.63, 95% CI: [0.72, 18.2]) nor between subject- and object-relatives (*β* = 0.34, *SE* = 0.39, *z* = 0.87, *p* = 0.43, Cohen’s *d* = 0.19, odds ratio = 1.41, 95% CI: [0.65, 3.04]).

#### 3.4.2 Variability and individual level performance in Mandarin

**FIGURE 7:**
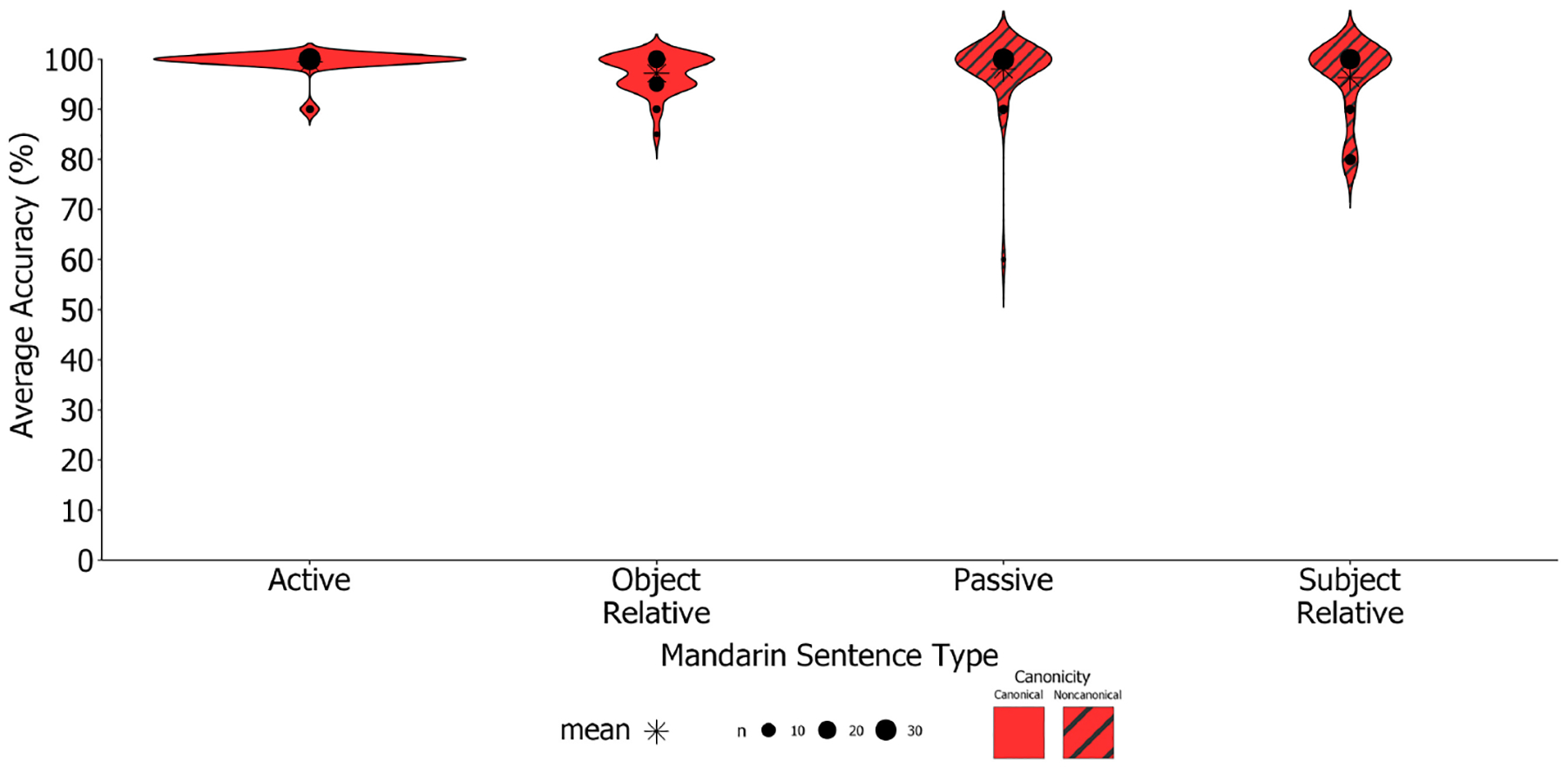
Study 2 distribution of performance across sentences in Mandarin. Non-canonical sentences (passives and subject-relatives) display a wider performance distribution, indicating increased variability in performance. *Indicates the mean accuracy for each sentence type in Mandarin while the size of the points indicates the number of participants performing at that level of accuracy.

Despite high overall performance on the *M-SOAP*, participants performed more variably on Mandarin passives and subject-relative constructions (both non-canonical; *SD*_*non*−*canonical*_ = 6.10) vs. canonical sentences (i.e., actives and object-relatives; *SD*_*canonical*_ = 2.57; see Figure 7 for distribution of performance).

**FIGURE 8:**
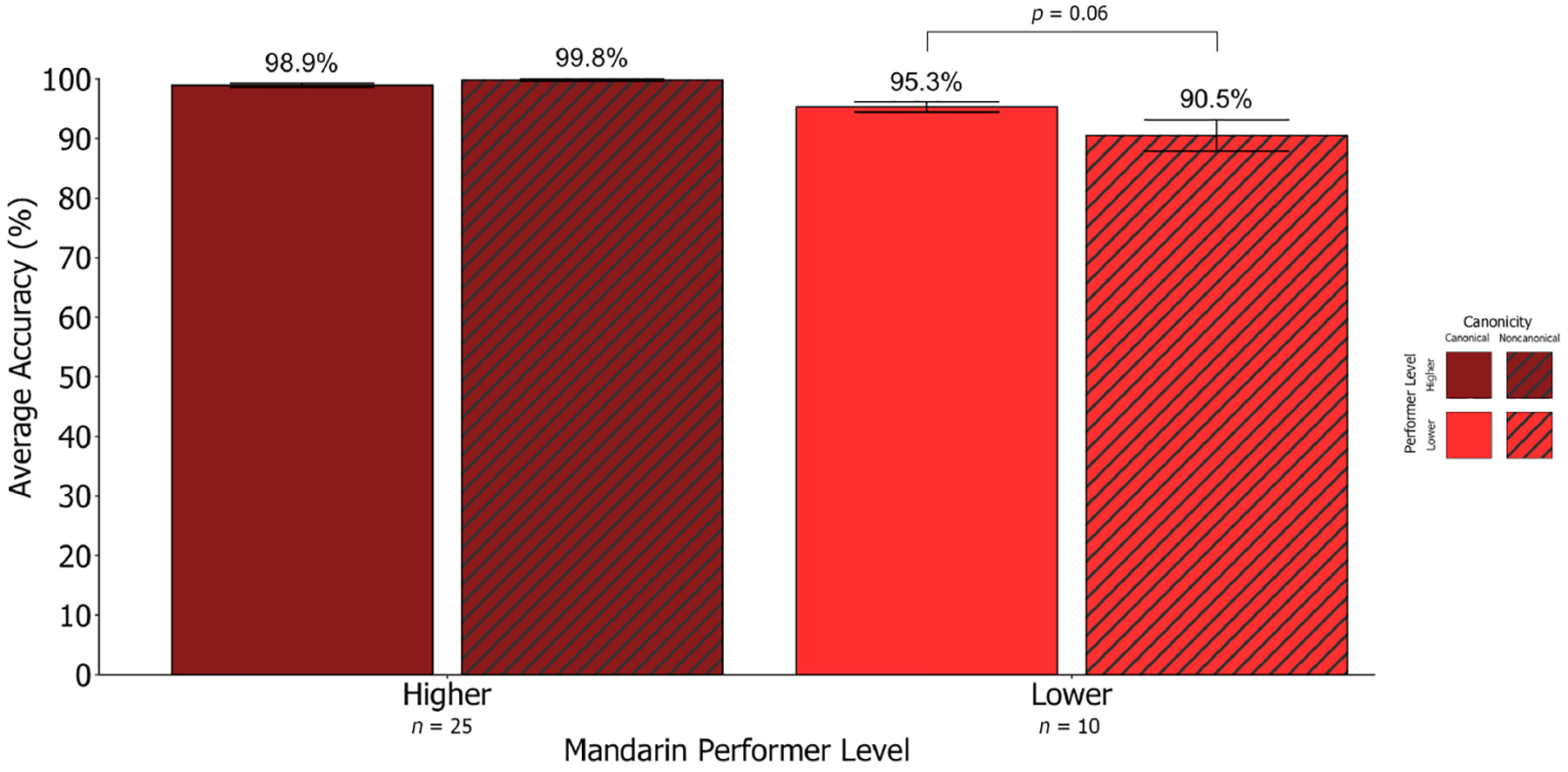
Study 2 average accuracy for Mandarin canonical and non-canonical sentences across performer levels. Note. Higher performers = above average (97.7%) performance across all Mandarin sentences (*n* = 25); lower performers = below average performance across all Mandarin sentences (*n* = 10).

Given the increased variability in performance for Mandarin non-canonical sentences, further analyses were conducted based on performer level (higher performers = above average [97.7%] performance on the overall M-SOAP [*n* = 25]; lower performers = below average performance on the overall M-SOAP [*n* = 10]). Using Wilcoxon Signed Rank Testing, we replicated findings from Study 1 where higher performers performed similarly across Mandarin canonical (98.9%) and non-canonical sentences (99.8%). Conversely, lower performers displayed lower accuracy on Mandarin non-canonical (90.5%) compared to canonical sentences (95.3%) where this difference approached significance (*Z* = −1.88, *p* = 0.06, *d* = 0.59, *n* = 10; see Figure 8). Lower performers’ worse non-canonical performance was driven by lower accuracy for passive sentences (93.0%) compared to active sentences (99.0%; *Z* = −2.04, *p* = 0.04, *d* = 0.65, *n* = 10). As in Study 1, performance did not significantly differ across subject- (88.0%) and object-relative (93.5%) sentences for lower performers (*Z* = 1.19, *p* = 0.24, *d* = 0.37, *n* = 10).

Finally, to examine whether age was associated with overall Mandarin performance, we collapsed participants’ overall *M-SOAP* accuracy scores across Studies 1 and 2 and conducted a Pearson correlation between overall performance and age. Results revealed a significant moderate negative correlation (*r* = −0.51, *p* < 0.0001), indicating that older participants performed worse on the *M-SOAP*. These findings are further interpreted in the Section 4.

## 4 General discussion

Our primary research goals were to examine the contested universal subject-relative processing advantage across the languages of bilingual speakers and use our sentence comprehension findings across simple and complex sentences to lend support to a theory or theories of sentence processing for bilingual speakers. In both of our studies, we used a sentence picture matching task to compare the comprehension performance between a pair of simple sentences (actives and passives), a pair of complex sentences (subject- and object-relatives), and more broadly across sentences with canonical (typical) and non-canonical (atypical) word order across English and Mandarin in bilingual speakers of varying proficiency levels.

Despite differences in their English language profiles, Study 1 and 2 participants demonstrated an English subject-relative and active sentence performance advantage, or more broadly a canonical advantage, as compared to object-relative and passive sentences, respectively. Study 1 participants were not dominant in English given lower reported English proficiency and less English exposure. In contrast, our bilingual participants from Study 2 demonstrated greater English dominance, greater English abilities, and greater English exposure. We thus show a robust pattern in English that is present regardless of proficiency profile.

These findings in English confirm prior monolingual English sentence processing findings that have consistently shown advantages for canonical subject-relative and active constructions in typical adults (Ferreira, 2003; Gibson, 1998; Gibson et al., 2005; Gordon et al., 2002; Grodner and Gibson, 2005; King and Just, 1991; King and Kutas, 1995; MacWhinney, 1982; Stromswold et al., 1996; Traxler et al., 2002; Wanner and Maratsos, 1978) and adults with aphasia (Cho-Reyes and Thompson, 2012; Grodzinsky, 1989; Horne et al., 2022; Love and Oster, 2002; Meyer et al., 2012; Pettigrew and Hillis, 2014). English has a rigid subject-verb-object (SVO) word order, consistently following properties characteristic of an SVO language. Consequently, the canonical, or typical, word order positions the agent before the patient. Given this rigidity, it is unsurprising that prior studies with monolingual English speakers and the present studies with bilinguals of varying English proficiencies align in their findings on English sentence comprehension across both simple and complex sentences. Moreover, for English, all discussed theoretical accounts of sentence processing based on syntactic structure and canonicity, as well as other theoretical accounts that were not explicitly discussed in the current paper (e.g., prominence-based accounts and memory-based accounts; see Table 1), all converge in predicting a subject-relative and active performance advantage over object-relatives and passives, respectively.

Our findings in Mandarin reveal a pattern that differs from the English findings but aligns with prior monolingual Mandarin sentence processing literature. In Mandarin, outcomes are inconsistent and less robust than in English (Chen et al., 2008; Gibson and Wu, 2013; Hsiao and Gibson, 2003; Lin and Bever, 2006; Lin and Garnsey, 2011; Packard et al., 2011; Qiao et al., 2012; Sun et al., 2016; Sung et al., 2016; Vasishth et al., 2013; Xu et al., 2019; Yang et al., 2010; Yang and Perfetti, 2006). While we did not find a significant difference in group-level accuracy between subject- and object-relatives, we note that subject-relative constructions exhibited greater variability in performance amongst older, more Mandarin dominant bilinguals and younger, more balanced bilinguals. Notably, individuals who were lower-performing in Mandarin particularly struggled with structures with non-canonical word order (subject-relatives and passives). Upon closer inspection, however, we noted that passives specifically drove these difficulties, while subject- and object-relative clauses showed similar performance patterns.

Overall, these findings from Mandarin lend some support to sentence processing theories that do not predict a universal subject-relative processing advantage, such as the top-down canonicity-based accounts, or other memory-based accounts (e.g., see Table 1). However, our results offer only partial support toward top-down canonicity-based accounts, given that these accounts would predict a *clear* group-level object-relative performance advantage that we did not see. Rather, we obtained increased performance variability on subject-relatives compared to object-relative constructions in Mandarin. Interestingly, findings from prior studies on sentence comprehension deficits in Chinese speakers with *aphasia* have, to our knowledge, all converged to show a greater difficulty with subject-relative constructions (Law and Leung, 1998; Law, 2000; Law and Leung, 2000; Wang and Thompson, 2016). This pattern from the aphasia literature is consistent with our observed pattern of greater subject-relative variability in unimpaired bilinguals. Perhaps top-down canonicity-based accounts do hold some merit, whereby individuals with aphasia show difficulty comprehending non-canonical structures and instead may rely on parsing a sentence based on the linear order of the noun phrases and assigning agent to the first encountered noun phrase (see Law, 2000, for further details). However, our results suggest that canonicity-based accounts (and related accounts, see Table 1) alone are not enough to capture cross-linguistic sentence comprehension, as we did not find overt group-level performance advantages for canonical structures across *both* English and Mandarin (as would be predicted by this account).

To explain the more comparable performance on subject- and object-relative sentences in Mandarin across our two studies, we look to theoretical accounts that make conflicting, rather than uniform (as in English) predictions for sentence comprehension in Mandarin. In Mandarin, theories based on syntactic structure predict that subject-relatives and actives should be easier to process, whereas canonicity-based theories predict that object-relatives and actives should be easier. Given these competing predictions, it is unsurprising that prior literature has reported mixed findings, including a subject-relative advantage (Lin and Bever, 2006; Vasishth et al., 2013), an object-relative advantage (Chen et al., 2008; Gibson and Wu, 2013; Hsiao and Gibson, 2003; Lin and Garnsey, 2011; Packard et al., 2011; Qiao et al., 2012; Sun et al., 2016; Sung et al., 2016; Vasishth et al., 2013; Xu et al., 2019; Yang et al., 2010; Yang and Perfetti, 2006), or no clear advantage (Vasishth et al., 2013). Overall, rather than attempting to determine which single theoretical account best explains these findings, we seek to reframe this approach as it may be more fruitful to consider how these theories interact in a unified manner. Thinking from structure-based (e.g., O’Grady, 1997), frequency-based (e.g., Mitchell et al., 1995), usage-based (Bybee, 2006; Goldberg, 2006; Ellis et al., 2016), canonicity-based (Bever, 1970; Swinney and Love, 1998), and memory-based (e.g., Gibson, 1998, 2000) perspectives collectively, object-relative constructions in Mandarin are structurally more complex and occur less frequently than subject-relative constructions in written and spoken corpora (Pu, 2007; Vasishth et al., 2013). At the same time, object-relatives may impose a lower memory load, as they align with the canonical agent–patient configuration that is semantically prototypical in natural language use (Hsiao and Gibson, 2003; Lin and Bever, 2006; Gibson and Wu, 2013). Importantly, canonicity reflects not only structural simplicity but also frequency-, experience-, and usage-based properties of language (MacDonald, 2013). Thus, the competing influences of structural complexity, frequency of exposure, and experiential entrenchment may counterbalance one another, resulting in the absence of a clear performance difference between subject- and object-relative constructions in Mandarin. Applying this integrated framework, the current findings suggest that Mandarin sentence processing reflects the joint influence of structural, memory-based, and experience-based mechanisms, rather than a single dominant account. Likewise, Vasishth et al. (2013) found a subject-relative advantage in two out of three Mandarin experiments they conducted and an object-relative advantage in the third experiment. They explain these discrepant findings in a similar manner, arguing that different accounts may operate in tandem across languages.

Furthermore, a broader explanation of the lack of a clear subject-relative or object-relative advantage in Mandarin may lie in Mandarin’s more flexible word order compared to English. Unlike English, where agent-theme SVO order is rigid, Mandarin speakers frequently encounter both agent-theme and themeagent structures since Mandarin is a topic-prominent language that has characteristics of SVO and SOV languages. Historically, Mandarin has been described as an SVO language that may be gradually changing to an SOV language (Li and Thompson, 1989). Increased exposure to sentences of varied order may allow Mandarin speakers to efficiently parse sentences with differing word order, leading to more balanced comprehension patterns across sentence constructions and a less pronounced top-down bias toward canonical forms. Despite the potential for language transfer effects, our bilingual participants performed similarly to monolinguals in each of their respective languages based on previous literature. This suggests that sentence comprehension and processing are governed by the intrinsic properties of each language, beyond cross-linguistic influence from a bilingual’s other language.

Potential crosslinguistic influences during sentence processing should not be ruled out based on the current findings. For example, evidence from cross-linguistic syntactic priming suggests that influence across English and Chinese at the structural level is possible (Li, 2025). It is possible that methodological choices made in the current studies reduced opportunities to observe cross-linguistic influence. Across both studies, participants demonstrated strong Mandarin performance, with accuracy approaching ceiling, especially in Study 2. Although participants in Study 2 were generally less Mandarin-dominant and reported greater English exposure compared to those in Study 1, they performed even better on the Mandarin task, a pattern best explained by age-related factors, with younger participants tending to perform better across the two studies. Additionally, while the *M-SOAP* stimuli were piloted for clarity and reliability, they were not specifically calibrated to avoid ceiling-level performance. This decision aligns with the test’s overarching goal of remaining accessible for healthy adults, as originally designed in Love and Oster’s (2002) English version, to later detect sentence-level deficits in clinical populations. Consequently, the high overall performance, combined with design features such as hearing each sentence twice and having unlimited response time, may have allowed participants sufficient opportunity to process and self-correct. Additionally, further noise may have been introduced given the uncontrolled timing variability across trials and participants in Study 1 that may have obscured potential sentence processing effects, although this variability occurred after responses had been made and not trial-internally. For these reasons, cross-linguistic influences that may have been visible along the time course of processing may have been obscured in the current studies, and conclusions regarding cross-linguistic convergence of processing mechanisms should therefore be made with caution.

Future research should supplement behavioral accuracy data with online processing measures such eye-tracking or electroencephalography (EEG). Offline accuracy measures, such as the ones collected here, while informative, may not capture the fine-grained temporal dynamics of sentence processing, particularly when performance is near ceiling. Subtle differences between canonical and non-canonical structures might have emerged in reaction time or online processing measures. More time-sensitive methodologies would enable the detection of subtle processing differences and provide a more comprehensive understanding of how age, language experience, and structural complexity might shape crosslinguistic influences in bilingual sentence comprehension.

Moving forward, research should also examine what underlying cognitive or linguistic constructs are driving any obtained differences in cross-linguistic comprehension. For example, to more thoroughly investigate the memory-based theoretical account of sentence processing, the working memory of participants should be measured and compared to performance (Akhavan et al., 2020; Gibson, 1998, 2000). Chen et al. (2008) found that working memory contributed to the processing of relative clauses in Mandarin, where individuals with lower working memory demonstrated increased processing time with subject-relatives while individuals with higher working memory demonstrated no difference in processing speeds across subject- and object-relatives.

Research in sentence processing and the underlying processing mechanisms is not only important to further our understanding of the human language processor but also holds utility in characterizing language deficits in various clinical populations, including individuals with aphasia, developmental language disorder (DLD), or dyslexia. Syntactic-based therapies such as the Treatment of Underlying Forms (TUF; Thompson and Shapiro, 2007) are used to improve sentence structure in agrammatic individuals with mild language impairments, where treatment starts with more complex sentence forms. The goal of TUF is for subsequent gains with complex sentence comprehension (e.g., passive sentences) to generalize to simpler sentences (e.g., active sentences) without being directly treated. Thus, it is vital to accurately classify the complexity and processing demands of Mandarin sentences, specifically relative clause structures, so that speech-language pathologists working with Mandarin-speaking individuals with aphasia can efficiently maximize treatment outcomes by treating more complex sentence forms, such that simpler syntactic forms will also benefit without direct intervention.

## 5 Conclusions

In the current paper, we examined bilingual sentence comprehension across English and Mandarin to evaluate the universality of the subject-relative clause advantage and to evaluate competing theoretical accounts of sentence processing. We asked whether Mandarin-English bilinguals would demonstrate converging sentence processing patterns across their two languages, consistent with structure-based theories, or diverging, language-specific patterns aligned with canonicity-based accounts. Two experiments using the *English and Mandarin SOAP Syntactic Batteries of Sentence Comprehension* tested comprehension of active, passive, subject-relative, and object-relative sentences through a picture-matching task.

Across studies, English results replicated prior monolingual findings, revealing a robust subject-relative and active processing advantage, even among less-proficient and later-exposed bilinguals, reflecting the rigidity of English’s canonical SVO word order and the reliance of even relatively recent adult English speakers on canonicity. In contrast, Mandarin findings showed greater variability, with no clear group-level difference between subject- and object-relative constructions, aligning with the mixed findings in the literature. Mandarin’s flexible word order may mitigate strong processing biases, contributing to the observed null group difference between subject- and object-relatives. These results suggest that sentence comprehension is shaped by an interaction of structural, canonicity-based, experience/usage/frequency-driven factors, rather than by a universal subject-relative advantage. Additionally, bilinguals’ performance largely paralleled monolingual norms, indicating that processing is governed by language-specific properties rather than cross-linguistic transfer effects.

Together, these findings highlight the importance of integrating multiple sentence processing accounts in order to model sentence processing. Methodologically, the high overall accuracy across both studies underscores the need for more sensitive and time-based measures to capture subtle processing differences. Clinically, these results have implications for assessing and treating sentence comprehension deficits in bilingual individuals with aphasia, emphasizing the need for language-specific approaches that account for both structural complexity and experience-based processing mechanisms.

## Supplementary Material

Rishi et al 2026 Supp File

The Supplementary Material for this article can be found online at: https://www.frontiersin.org/articles/10.3389/flang.2025.1703230/full#supplementary-material

## Figures and Tables

**TABLE 1:** Summary of sentence processing theories and their predictions for processing simple and complex sentences across English and Mandarin.

Sentence processing theory	Description	English sentence prediction	Mandarin sentence prediction
Structural-based accounts (e.g., O’Grady, 1997)	Sentence processing difficulty is determined by the number of syntactic nodes separating dependent elements, with greater structural distance leading to increased processing cost.	SR easier than OR Actives easier than passives	SR easier than OR Actives easier than passives
Frequency, Experience, and Usage-based accounts (e.g., Mitchell et al., 1995; Bybee, 2006; Goldberg, 2006; Ellis et al., 2016; Pu, 2007)	The ease of parsing a structure depends on one’s experience and usage of such structures, including the frequency with which structures occur and are used by a speaker. More frequently encountered and/or used structures are processed more efficiently.		
Prominence-based accounts (e.g., Mak et al., 2006; O’Grady, 2011; Lin, 2018)	Structures are easier to process when they align with discourse prominence hierarchies, such as topic-hood, animacy, and givenness.		
Top-down Canonicity-based accounts (e.g., Bever, 1970; Swinney and Love, 1998)	Sentence processing is guided by strong expectations for canonical word order (e.g., SVO in English), making structures that conform to this default easier to process, while non-canonical structures require additional reanalysis and thus incur greater processing costs.		OR easier than SR Actives easier than passives
Memory-based accounts (e.g., Gibson, 1998, 2000)	Sentence processing difficulty increases with greater integration cost, which depends on the distance between syntactically dependent elements, and memory cost as determined by the number of intervening discourse referents that must be maintained in working memory.		

SR, subject-relative; OR, object-relative.

**TABLE 2:** Study 1 participant demographics, language, and cognitive characteristics (*n* = 18).

Participant characteristics	Mean	SD	Range
Age	76.81	4.08	70–86
Years of formal education	15.61	1.72	12–18
Cognitive performance (*MoCA-BJ*; out of 30)^a^	24.78	3.23	18–29
Age of immigration to United States^b^	68.56	4.98	61–76
Language dominance score^c^	0.47	0.19	0.12–0.79
**English characteristics**			
Age of first exposure (years)	32.38	23.45	0–68
Current exposure (%)	11.21	9.93	0–40
Self-reported speaking (out of 10)	2.31	1.35	0–5
Self-reported comprehension (out of 10)	2.19	1.68	0–7
Self-reported reading (out of 10)	2.00	2.19	0–7
Verbal fluency–animals (raw score)	7.50	5.92	0–19
Verbal fluency–groceries (raw score)	9.44	4.44	1–16
**Mandarin characteristics**			
Age of first exposure (years)^d^	4.82	2.16	0–10
Current exposure (%)	88.80	9.57	60–100
Self-reported speaking (out of 10)	8.15	1.03	7–10
Self-reported comprehension (out of 10)	8.32	0.81	7–10
Self-reported reading (out of 10)	8.41	1.06	7–10
Verbal fluency–animals (raw score)	16.11	3.14	10–21
Verbal fluency–groceries (raw score)	16.50	3.93	8–24

a All participants performed within 1.5 standard deviations of the typical average on the Beijing version of the Montreal Cognitive Assessment (MoCA-BJ) for their age range (Hong et al., 2022).

b All 18 participants immigrated to the United States.

c Language dominance is interpreted on a scale from −1 (English-dominant) to 1 (Mandarin-dominant). 0 indicates a balanced bilingual. Language dominance for Study 1 was calculated by averaging across Language, Experience, and Proficiency Questionnaire (LEAP-Q) self-ratings for speaking, comprehension, and reading, as well as the groceries verbal fluency score. One participant did not have a language dominance score calculated as LEAP-Q self-ratings were not obtained.

d Age of first exposure to Mandarin was not as uniformly near birth despite Mandarin being reported as all participants’ L1, given that Mandarin speakers, including those in the Study 1 sample, are frequently, but are not always exposed to another local Chinese dialect first before being exposed to Mandarin in school. Chinese dialects share linguistic properties with Mandarin, and thus many Mandarin speakers report Mandarin as their L1.

**TABLE 3:** Study 2 participant demographics, language, and cognitive characteristics (*n* = 35).

Participant characteristics	Mean	SD	Range
Age	42.43	20.76	18–85
Years of formal education	16.54	3.17	9–26
Cognitive performance (*MoCA/MoCA-ChLA*; out of 30)^a^	28.23	1.72	24–30
Age of immigration to United States^b^	23.71	11.10	8–51
Language dominance score^c^	0.02	0.32	−0.60 to 0.46
**English characteristics**			
Age of first exposure (years)	8.26	5.25	0–24
Current exposure (%)	47.57	29.47	10–95
Self-reported speaking (out of 10)	7.51	1.88	1–10
Self-reported comprehension (out of 10)	7.66	1.85	1–10
Self-reported reading (out of 10)	7.83	1.87	1–10
*MINT-Sprint* score (%)	76.64	15.06	41.25–100
**Mandarin characteristics**			
Age of first exposure (years)	2.77	3.73	0–14
Current exposure (%)^d^	46.71	28.72	5–90
Self-reported speaking (out of 10)	8.49	1.88	3–10
Self-reported comprehension (out of 10)	8.66	1.86	3–10
Self-reported reading (out of 10)	8.46	2.06	3–10
*MINT-Sprint* score (%)	74.75	16.90	27.5–95

a All participants performed within 1.5 standard deviations of the typical average on the English Montreal Cognitive Assessment (MoCA) or the Chinese-Language Los Angeles Version of the MoCA (MoCA-ChLA; Zheng et al., 2012) for their age range according to language-specific standards (Nasreddine et al., 2005; Hong et al., 2022; Wei et al., 2024).

b 24 participants immigrated to the United States (68.7% of the sample).

c Language dominance is interpreted on a scale from −1 (English-dominant) to 1 (Mandarin-dominant). 0 indicates a balanced bilingual. Language dominance for Study 2 was calculated by averaging across Language, Experience, and Proficiency Questionnaire (LEAP-Q; Marian et al., 2007; Blumenfeld et al., 2017) self-ratings for speaking, comprehension, and reading, current exposure, as well as Multilingual Naming Test-Sprint Version (MINT-Sprint; Garcia and Gollan, 2022) scores.

d Current exposure reported for Study 2 was a composite exposure to Chinese (including all dialects).

## Data Availability

The datasets presented in this study can be found in online repositories. The names of the repository/repositories and accession number(s) can be found below: Open Science Framework: https://osf.io/h5g7w/?view_only=1432aed8289b44c4b7c04e4a51b4e254.

## Footnotes

3 In examples 4a, b, prenominal modifier phrases end with the morpheme 的 *de* linking the modifying phrase to the head noun. In doing so, 的 *de* indicates the right edge of a modifying phrase and the left edge of the head noun and is often glossed as a relative clause marker (REL) in relative clauses in Mandarin although it also occurs in other constructions with modifier + noun phrases.

4 Trace, gap, and extracted argument position are used interchangeably here to refer to the same entity since some of the existing literature use one term or the other. For simplicity, the term trace will be used henceforth.

5 Three of the included participants reported having a head injury in their adult life due to a fall (*n* = 2) or laboratory explosion (*n* = 1), but none reported loss of consciousness after the injury.

6 Since many words in Mandarin are compound words, sentence length was measured by Chinese character count where each character corresponds to a syllable (as opposed to word length which is typically used for measuring length in English).

7 We also obtained a significant 3-way interaction between Language × Canonicity × Relative Clause (*χ^2^*[1,19] = 4.36, *p* = 0.04, *β*_*raw*_ = 1.59, *SE* = 0.76, Wald *Z* = 2.09) that is not relevant to the current study and thus will not be discussed further. Results were generally consistent whether sentence length was included as a covariate measured in word count (English)/character count (Mandarin) or length in seconds (reported here). Findings were also similar when no covariates were included, with the exception that the difference between participants’ Mandarin performance on canonical and non-canonical sentences did not reach significance (*p* = 0.07) without any covariates included.

8 Wald *Z* and Wald *χ*^2^ statistics may appear discrepant, as they test related but distinct hypotheses where Wald *Z* assesses individual coefficients while Wald *χ*^2^ tests the effect of the term as a whole using the full variance-covariance matrix.

9 For Mandarin, a composite measure of current exposure to Chinese (including all dialects) was calculated, as exposure to any Chinese language was assumed to influence Mandarin skills due to overlapping linguistic characteristics, particularly in grammar and syntax. Such detailed dialect exposure information was lacking for all participants in Study 1.

10 We also obtained a significant main effect of relative clause (*χ^2^*[1,35] = 5.50, *p* = 0.02, *β*_*raw*_ = 0.68, *SE* = 1.24, Wald *Z* = 0.55; *M*_*Relative Clause*_ (*SR/OR*) = 96.3%, *M*_*No Relative Clause*(*A/P*)_ = 97.6%) that is not relevant to the current study and thus will not be discussed further. As in Study 1, results were generally consistent whether sentence length was included as a covariate measured in word count (English)/character count (Mandarin) or length in seconds (reported here). The only exception was that the main effect of Language did not approach significance when sentence length measured in word/syllable count was used as a covariate (*p* = 0.12) instead of sentence length measured in seconds (reported here). Findings were also similar when no covariates were included.
