# Supplementary material for: Spoken sentence comprehension in Mandarin-English bilinguals: a case against the universal processing advantage of subject-relatives
Source: Front Lang Sci. Author manuscript; Available in PMC 2026 Feb 13. (PMC12900535; doi:10.3389/flang.2025.1703230)
Supplement: Rishi et al 2026 Supp File [file NIHMS2144727-supplement-Rishi_et_al_2026_Supp_File.pdf]

Appendix

Appendix 1: *English SOAP* (*E-SOAP*) Stimulus Sentences, where A = active, P = passive, SR = subject-relative, and OR = object-relative

Note. For Study 2, in sentences containing the characters *cowboy* and *Indian* (items 3, 8, 19, and 33), *cowboy* was replaced with *painter* and *Indian* was replaced with *gardener* to improve cultural congruence.

Practice
1. The old man with the beard leads the little boy.
2. The woman in the bathing suit dries the child.
3. The teacher that scolds the student has black hair.
4. The nurse on the 5th floor examines the doctor.
5. The policeman that threatens the man has black hair.

Test
1. (P) The doctor with blonde hair is questioned by the soldier.
2. (SR) The man that pushes the boy is wearing a red shirt.
3. (A) The thin cowboy with the blue pants captures the Indian.
4. (A) The young boy with the brown hair grabs the man.
5. (OR) The doctor that the bedridden patient accuses has black hair.
6. (P) The boy with the brown hair is grabbed by the man.
7. (A) The little boy with the big book yells at the soldier.
8. (SR) The cowboy that captures the Indian has on blue pants.
9. (OR) The boy that the girl chases is wearing a green shirt.
10. (OR) The man that the young boy grabs has brown hair.
11. (SR) The man that records the young woman has brown hair.
12. (P) The boy in the green shirt is chased by the girl.
13. (A) The girl with blonde hair photographs the nurse.
14. (P) The man in the red shirt is pushed by the boy.
15. (A) The little boy in the blue shirt instructs the teacher.
16. (SR) The doctor that accuses the bedridden patient has black hair.
17. (OR) The teacher that the little boy instructs has on a blue shirt.
18. (SR) The girl that photographs the nurse with the camera is blonde.
19. (P) The cowboy with blue pants is captured by the Indian.
20. (OR) The soldier that the little boy yells at has black hair.
21. (A) The young doctor with blonde hair questions the soldier.
22. (SR) The man that grabs the little boy has brown hair.
23. (A) The man in the red shirt pushes the little boy.
24. (A) The man with brown hair records the young woman.
25. (SR) The soldier that yells at the small boy has black hair.
26. (A) The bedridden patient with the black hair accuses the doctor.
27. (P) The boy with black hair is yelled at by the soldier.
28. (SR) The soldier in uniform that questions the doctor has blonde hair.
29. (SR) The teacher that instructs the boy is wearing a blue shirt.
30. (OR) The girl that the nurse photographs with the camera is blonde.
31. (OR) The man that the young woman records has brown hair.

32. (P) The boy in the blue shirt is instructed by the teacher.
33. (OR) The cowboy that the Indian captures has on blue pants.
34. (OR) The man that the boy pushes is wearing a red shirt.
35. (P) The patient with black hair is accused by the doctor.
36. (OR) The soldier in uniform that the doctor questions has blonde hair.
37. (A) The girl in the green shirt chases the small boy.
38. (P) The man with brown hair is recorded by the woman.
39. (P) The girl is photographed by the nurse with blonde hair.
40. (SR) The boy that chases the girl is wearing a green shirt.

Appendix 2: *Mandarin SOAP* (*M-SOAP*) Stimulus Sentences from Study 1, where A = active, P = passive, SR = subject-relative, and OR = object-relative. Each item is shown as follows:

first line - traditional Chinese script
second line - simplified Chinese script
third line - Pinyin (romanization)
fourth line - gloss
fifth line - English translation and sentence type

Note. Due to an oversight during translation and adaptation from English to Mandarin sentences in Study 1, three Mandarin sentences (items 30, 36, and 39) contained local modifiers in the middle of the sentence. Accuracy was not affected, as performance on these items was above or within one standard deviation below the overall mean accuracy for all Mandarin sentences in Study 1.

Practice

(1) 有 鬍子 的 老 男人 帶著 小 男孩。
有 胡子 的 老 男人 带着 小 男孩。
yǒu húzǐ de lǎo nánrén dàizhe xiǎo nánhái.
have beard REL old man lead-DUR small boy
The old man with the beard leads the little boy. (A)

(2) 穿著 游泳 衣 的 女人 把 小 孩子 擦乾。
穿着 游泳 衣 的 女人 把 小 孩子 擦干。
chuānzhe yóuyǒng yī de nǚrén bǎ xiǎo háizǐ cāgān.
wear-DUR swim clothes REL woman BA small child dry.
The woman in the bathing suit dries the child. (A)

(3) 責罵 學生 的 老師 有 一頭 黑髮。
责骂 学生 的 老师 有 一头 黑发。
zémà xuéshēng de lǎoshī yǒu yītóu hēifā.
scold student REL teacher have one-CL black hair
The teacher that scolds the student has black hair. (SR)

(4) 在 五 樓 的 護士 檢查 醫生。
在 五 楼 的 护士 检查 医生。
zài wǔ lóu de hùshì jiǎnchá yīshēng.
at five floor REL nurse examine doctor

The nurse on the fifth floor examines the doctor. (A)

(5) 威脅 男人 的 警察 有 一頭 黑髮。
威胁 男人 的 警察 有 一头 黑发。
wēixié nánrén de jǐngchá yǒu yītóu hēifā.
threaten man REL police have one-CL black hair.
The policeman that threatens the man has black hair. (SR)

Test

(1) 金髮 的 醫生 被 士兵 質問。
金发 的 医生 被 士兵 质问。
jīnfā de yīshēng bèi shìbīng zhíwèn.
blonde REL doctor PAS soldier questioned
The doctor with blonde hair is questioned by the soldier. (P)

(2) 推 男孩 的 男人 穿著 紅色 上衣。
推 男孩 的 男人 穿着 红色 上衣。
tuī nánhái de nánrén chuānzhe hóngsè shàngyī.
push boy REL man wear-DUR red shirt
The man that pushes the boy is wearing a red shirt. (SR)

(3) 穿著 藍色 褲子 的 瘦 牛仔 逮捕 印地安人。
穿着 蓝色 裤子 的 瘦 牛仔 逮捕 印地安人。
chuānzhe lánsè kùzi de shòu niúzǎi dàibǔ yìndìānrén.
wear-DUR blue pants REL thin cowboy capture Indian
The thin cowboy with the blue pants captures the Indian. (A)

(4) 棕髮 的 年輕 男孩 抓著 男人。
棕发 的 年轻 男孩 抓着 男人。
zōngfà de niánqīng nánhái zhuāzhe nánrén.
brown hair REL young boy grab-DUR man
The young boy with the brown hair grabs the man. (A)

(5) 被 病人 指控 的 醫生 有 一頭 黑髮。
被 病人 指控 的 医生 有 一头 黑发。
bèi bìngrén zhǐkòng de yīshēng yǒu yītóu hēifà.
PAS patient accuse REL doctor has one-CL black hair
The doctor that the patient accuses has black hair. (OR)

(6) 棕髮 的 男孩 被 男人 抓住。
棕发 的 男孩 被 男人 抓住。
zōngfà de nánhái bèi nánrén zhuāzhù.
brown hair REL boy PAS man grab-hold on
The boy with the brown hair is grabbed by the man. (P)

(7) 拿著 一本 大 書 的 小 男孩 向 士兵 吼叫。

拿着 一本 大 书 的 小 男孩 向 士兵 吼叫。
názhe yīběn dà shū de xiǎo nánhái xiàng shìbīng hǒujiào.
carry-DUR one-CL big book REL little boy towards soldier shout-call
The little boy with the big book yells at the soldier. (A)

(8) 逮捕 印地安人 的 牛仔 穿著 藍 褲子。
逮捕 印地安人 的 牛仔 穿着 蓝 裤子。
dàibǔ yìndiānrén de niúzǎi chuānzhe lán kùzi.
capture Indian REL cowboy wear-DUR blue pants
The cowboy that captures the Indian has on blue pants. (SR)

(9) 被 女孩 追 的 男孩 穿著 綠色 上衣。
被 女孩 追 的 男孩 穿着 绿色 上衣。
bèi nǚhái zhuī de nánhái chuānzhe lǜsè shàngyī.
PAS girl chase REL boy wear-DUR green shirt
The boy that the girl chases is wearing a green shirt. (OR)

(10) 被 年輕 男孩 抓著 的 男人 有 一頭 棕髮。
被 年轻 男孩 抓着 的 男人 有 一头 棕发。
bèi niánqīng nánhái zhuāzhe de nánrén yǒu yītóu zōngfà.
PAS young boy grab-DUR REL man has one-CL brown hair
The man that the young boy grabs has brown hair. (OR)

(11) 替 年輕 女人 錄影 的 男人 有 一頭 棕髮。
替 年轻 女人 录影 的 男人 有 一头 棕发。
tì niánqīng nǚrén lùyǐng de nánrén yǒu yītóu zōngfà.
for young woman record REL man has one-CL brown hair
The man that records the young woman has brown hair. (SR)

(12) 穿著 綠色 上衣 的 男孩 被 女孩 追。
穿着 绿色 上衣 的 男孩 被 女孩 追。
chuānzhe lǜsè shàngyī de nánhái bèi nǚhái zhuī.
wear-DUR green shirt REL boy PAS girl chase
The boy in the green shirt is chased by the girl. (P)

(13) 金髮 的 女孩 替 護士 拍照。
金发 的 女孩 替 护士 拍照。
jīnfà de nǚhái tì hùshi pāizhào.
blonde REL girl for nurse photograph
The girl with blonde hair photographs the nurse. (A)

(14) 穿著 紅色 上衣 的 男人 被 男孩 推。
穿着 红色 上衣 的 男人 被 男孩 推。
chuānzhe hóngsè shàngyī de nánrén bèi nánhái tuī.
wear-DUR red shirt REL man PAS boy push
The man in the red shirt is pushed by the boy. (P)

(15) 穿著 藍色 上衣 的 小 男孩 指示 老師。
穿着 蓝色 上衣 的 小 男孩 指示 老师。
chuānzhe lánsè shàngyī de xiǎo nánhái zhǐshì lǎoshī.
wear-DUR blue shirt REL little boy instruct teacher
The little boy in the blue shirt instructs the teacher. (A)

(16) 指控 病人 的 醫生 有 一頭 黑髮。
指控 病人 的 医生 有 一头 黑发。
zhǐkòng bìngrén de yīshēng yǒu yītóu hēifà.
accuse patient REL doctor has one-CL black hair
The doctor that accuses the patient has black hair. (SR)

(17) 被 小 男孩 指示 的 老師 穿著 藍色 上衣。
被 小 男孩 指示 的 老师 穿着 蓝色 上衣。
bèi xiǎo nánhái zhǐshì de lǎoshī chuānzhe lánsè shàngyī.
PAS little boy instruct REL teacher wear-DUR blue shirt
The teacher that the little boy instructs has on a blue shirt. (OR)

(18) 持 相機 拍照 護士 的 女孩 是 金髮 的。
持 相机 拍照 护士 的 女孩 是 金发 的。
chí xiàngjī pāizhào hùshi de nǚhái shì jīnfà de.
hold camera photograph nurse REL girl is blonde POS
The girl that photographs the nurse with the camera is blonde. (SR)

(19) 穿著 藍色 褲子 的 牛仔 被 印地安人 逮捕。
穿着 蓝色 裤子 的 牛仔 被 印地安人 逮捕。
chuānzhe lánsè kùzi de niúzǎi bèi yìndìānrén dàibǔ.
wear-DUR blue pants REL cowboy PAS Indian capture
The cowboy with blue pants is captured by the Indian. (P)

(20) 被 小 男孩 吼叫 的 士兵 有 一頭 黑髮。
被 小 男孩 吼叫 的 士兵 有 一头 黑发。
bèi xiǎo nánhái hǒujiào de shìbīng yǒu yītóu hēifà.
PAS little boy shout-call REL soldier has one-CL black hair
The soldier that the little boy yells at has black hair. (OR)

(21) 金髮 的 年輕 醫生 質問 士兵。
金发 的 年轻 医生 质问 士兵。
jīnfǎ de niánqīng yīshēng zhíwèn shìbīng.
blonde REL young doctor question soldier
The young doctor with blonde hair questions the soldier. (A)

(22) 抓住 小 男孩 的 男人 有 一頭 棕髮。
抓住 小 男孩 的 男人 有 一头 棕发。
zhuāzhù xiǎo nánhái de nánrén yǒu yītóu zōngfà.
grab-hold on little boy REL man has one-CL brown hair

The man that grabs the little boy has brown hair. (SR)

(23) 穿著 紅色 上衣 的 男人 推 小 男孩。
穿着 红色 上衣 的 男人 推 小 男孩。
chuānzhe hóngsè shàngyī de nánrén tuī xiǎo nánhái.
wear-DUR red shirt REL man push little boy
The man in the red shirt pushes the little boy. (A)

(24) 棕髮 的 男人 替 年輕 女人 錄影。
棕发 的 男人 替 年轻 女人 录影。
zōngfà de nánrén tì niánqīng nǚrén lùyǐng.
brown hair REL man for young woman record
The man with the brown hair records the young woman. (A)

(25) 向 小 男孩 吼叫 的 士兵 有 一頭 黑髮。
向 小 男孩 吼叫 的 士兵 有 一头 黑发。
xiàng xiǎo nánhái hǒujiào de shìbīng yǒu yītóu hēifà.
towards little boy shout-call REL soldier has one-CL black hair.
The soldier that yells at the small boy has black hair. (SR)

(26) 黑髮 的 病人 指控 醫生。
黑发 的 病人 指控 医生。
hēifà de bìngrén zhǐkòng yīshēng.
black hair REL patient accuse doctor
The patient with the black hair accuses the doctor. (A)

(27) 黑髮 的 男孩 被 士兵 吼叫。
黑发 的 男孩 被 士兵 吼叫。
hēifà de nánhái bèi shìbīng hǒujiào.
black hair REL boy PAS soldier shout-call
The boy with black hair is yelled at by the soldier. (P)

(28) 穿著 制服 質問 醫生 的 士兵 有 一頭 金髮。
穿着 制服 质问 医生 的 士兵 有 一头 金发。
chuānzhe zhìfú zhìwèn yīshēng de shìbīng yǒu yītóu jīnfǎ.
wear-DUR uniform question doctor REL soldier has one-CL blonde hair
The soldier in uniform that questions the doctor has blonde hair. (SR)

(29) 指示 男孩 的 老師 穿著 藍色 上衣。
指示 男孩 的 老师 穿着 蓝色 上衣。
zhǐshì nánhái de lǎoshī chuānzhe lánsè shàngyī.
instruct boy REL teacher wear-DUR blue shirt
The teacher that instructs the boy is wearing a blue shirt. (SR)

(30) 被 護士 以 相機 拍照 的 女孩 是 金髮 的。
被 护士 以 相机 拍照 的 女孩 是 金发 的。
bèi hùshi yǐ xiàngjī pāizhào de nǚhái shì jīnfà de.

PAS nurse with camera photograph REL girl is blonde POS
The girl that the nurse photographs with the camera is blonde. (OR)

(31) 被 年輕 女人 錄影 的 男人 有 一頭 棕髮。
被 年轻 女人 录影 的 男人 有 一头 棕发。
bèi niánqīng nǚrén lùyǐng de nánrén yǒu yītóu zōngfà.
PAS young woman record REL man has one-CL brown hair
The man that the young woman records has brown hair. (OR)

(32) 穿著 藍色 上衣 的 男孩 被 老師 指示。
穿着 蓝色 上衣 的 男孩 被 老师 指示。
chuānzhe lánsè shàngyī de nánhái bèi lǎoshī zhǐshì.
wear-DUR blue shirt REL boy PAS teacher instruct
The boy in the blue shirt is instructed by the teacher. (P)

(33) 被 印地安人 逮捕 的 牛仔 穿著 藍色 褲子。
被 印地安人 逮捕 的 牛仔 穿着 蓝色 裤子。
bèi yìndìānrén dàibǔ de niúzǎi chuānzhe lánsè kùzi.
PAS Indian capture REL cowboy wear-DUR blue pants
The cowboy that the Indian captures has on blue pants. (OR)

(34) 被 小 男孩 推 的 男人 穿著 紅色 上衣。
被 小 男孩 推 的 男人 穿着 红色 上衣。
bèi xiǎo nánhái tuī de nánrén chuānzhe hóngsè shàngyī.
PAS little boy push REL man wear-DUR red shirt
The man that the boy pushes is wearing a red shirt. (OR)

(35) 黑髮 的 病人 被 醫生 指控。
黑发 的 病人 被 医生 指控。
hēifà de bìngrén bèi yīshēng zhǐkòng.
black hair REL patient PAS doctor accuse
The patient with black hair is accused by the doctor. (P)

(36) 被 醫生 質問 穿著 制服 的 士兵 有 一頭 金髮。
被 医生 质问 穿着 制服 的 士兵 有 一头 金发。
bèi yīshēng zhìwèn chuānzhe zhìfú de shìbīng yǒu yītóu jīnfǎ.
PAS doctor question wear-DUR uniform REL soldier has one-CL blonde hair
The soldier in uniform that the doctor questions has blonde hair. (OR)

(37) 穿著 綠色 上衣 的 女孩 追 小 男孩。
穿着 绿色 上衣 的 女孩 追 小 男孩。
chuānzhe lǜsè shàngyī de nǚhái zhuī xiǎo nánhái.
wear-DUR green shirt REL girl chase little boy
The girl in the green shirt chases the small boy. (A)

(38) 棕髮 的 男人 被 女人 錄影。

棕发 的 男人 被 女人 录影。
zōngfà de nánrén bèi nǚrén lùyǐng.
brown hair REL man PAS woman record
The man with brown hair is recorded by the woman. (P)

(39) 女孩 被 金髮 的 護士 拍照。
女孩 被 金发 的 护士 拍照。
nǚhái bèi jīnfà de hùshi pāizhào.
girl PAS blonde REL nurse photograph
The girl is photographed by the nurse with blonde hair. (P)

(40) 追 女孩 的 男孩 穿著 綠色 上衣。
追 女孩 的 男孩 穿着 绿色 上衣。
zhuī nǚhái de nánhái chuānzhe lǜsè shàngyī.
chase girl REL boy wear-DUR green shirt
The boy that chases the girl is wearing a green shirt. (SR)

Appendix 3: *Mandarin SOAP* (*M-SOAP*) Stimulus Sentences from Study 2, where A = active, P = passive, SR = subject-relative, OR = object-relative without sentence-initial *bèi* marker, and *bèi*-OR = object-relative with sentence-initial *bèi* marker. Each item is shown as follows:

first line - traditional Chinese script
second line - simplified Chinese script
third line - Pinyin (romanization)
fourth line - gloss
fifth line - English translation and sentence type

Practice

(1) 有著 鬍子 的 老 先生 牽著 小 男孩。
有着 胡子 的 老 先生 牵着 小 男孩。
yǒuzhe húzǐ de lǎo xiānshēng qiānzhe xiǎo nánhái.
have-DUR beard REL old man lead small boy
The old man with the beard leads the little boy. (A)

(2) 穿著 泳 衣 的 女人 把 小 孩子 擦乾。
穿着 泳 衣 的 女人 把 小 孩子 擦干。
chuānzhe yǒng yī de nǚrén bǎ xiǎo háizǐ cāgān.
wear-DUR swim clothes REL woman BA small child dry
The woman in the bathing suit dries the child. (A)

(3) 被 老師 教訓 的 學生 有著 一頭 黑髮。
被 老师 教训 的 学生 有着 一头 黑发。
bèi lǎoshī jiàoxùn de xuéshēng yǒuzhe yītóu hēifā.
PAS teacher scold REL student have-DUR one-CL black hair
The student being scolded by the teacher has black hair. (OR)

(4) 五樓 的 護士 為 醫生 做 檢查。

五楼 的 护士 为 医生 做 检查。
wŭlóu de hùshì wéi yīshēng zuò jiǎnchá.
five floor REL nurse for doctor do examination
The nurse on the fifth floor does an examination for the doctor. (A)

(5) 被 警察 威脅 的 男人 有著 一頭 黑髮。
被 警察 威胁 的 男人 有着 一头 黑发。
bèi jǐngchá wēixié de nánrén yǒuzhe yītóu hēifā.
PAS police threaten REL man have-DUR one-CL black hair
The man being threatened by the police officer has black hair. (OR)

Test

(1) 有著 一頭 黑髮 的 醫生 指責 那個 病人。
有着 一头 黑发 的 医生 指责 那个 病人。
yǒuzhe yītóu hēifà de yīshēng zhǐzé nàgè bìngrén.
have-DUR one-CL black hair REL doctor accuse that-CL patient
The doctor with black hair accuses that patient. (A)

(2) 穿著 藍色 褲子 的 畫家 被 那個 園丁 抓到。
穿着 蓝色 裤子 的 画家 被 那个 园丁 抓到。
chuānzhe lánsè kùzi de huàjiā bèi nàgè yuándīng zhuādào.
wear-DUR blue pants REL painter PAS that-CL gardener capture-arrive
The painter with the blue pants is captured by that gardener. (P)

(3) 被 護士 用 相機 拍 的 那個 女孩 有著 一頭 金髮。
被 护士 用 相机 拍 的 那个 女孩 有着 一头 金发。
bèi hùshì yòng xiàngjī pāi de nàgè nǚhái yǒuzhe yītóu jīnfà.
PAS nurse use camera photograph REL that-CL girl have-DUR one-CL blonde hair
That girl that is photographed by the nurse using the camera has blonde hair. (bèi-OR)

(4) 有著 一頭 棕髮 的 男人 被 那個 女人 錄影。
有着 一头 棕发 的 男人 被 那个 女人 录影。
yǒuzhe yītóu zōngfà de nánrén bèi nàgè nǚrén lùyǐng
have-DUR one-CL brown hair REL man PAS that-CL woman record
The man with brown hair is recorded by that woman. (P)

(5) 被 小 男孩 推 的 那個 男人 穿著 紅色 上衣。
被 小 男孩 推 的 那个 男人 穿着 红色 上衣。
bèi xiǎo nánhái tuī de nàge nánrén chuānzhe hóngsè shàngyī.
PAS small boy push REL that-CL man wear-DUR red shirt
That man that is pushed by the little boy is wearing a red shirt. (bèi-OR)

(6) 女孩 追著 的 那個 男孩 穿著 綠色 上衣。
女孩 追着 的 那个 男孩 穿着 绿色 上衣。

nǚhái zhuīzhe de nàge nánhái chuānzhe lǜsè shàngyī.
girl chase-DUR REL that-CL boy wear-DUR green shirt
That boy that the girl chases is wearing a green shirt. (OR)

(7) 抓著 小 男孩 的 那個 男人 有著 一頭 棕髮。
抓着 小 男孩 的 那个 男人 有着 一头 棕发。
zhuāzhe xiǎo nánhái de nàge nánrén yǒuzhe yītóu zōngfà.
grab-DUR small boy REL that-CL man has-DUR one-CL brown hair
That man that grabs the little boy has brown hair. (SR)

(8) 有著 一頭 金髮 的 士兵 質問 那個 醫生。
有着 一头 金发 的 士兵 质问 那个 医生。
yǒuzhe yītóu jīnfǎ de shìbīng zhíwèn nàge yīshēng.
has-DUR one-CL blonde hair REL soldier question that-CL doctor
The soldier with blonde hair questions that doctor. (A)

(9) 士兵 大吼 的 那個 小 男孩 有著 一頭 黑髮。
士兵 大吼 的 那个 小 男孩 有着 一头 黑发。
shìbīng dàhǒu de nàgè xiǎo nánhái yǒuzhe yītóu hēifà.
soldier yell REL that-CL small boy has-DUR one-CL black hair
That little boy that the soldier yells at has black hair. (OR)

(10) 有著 一頭 黑髮 的 醫生 被 那個 病人 指責。
有着 一头 黑发 的 医生 被 那个 病人 指责。
yǒuzhe yītóu hēifà de yīshēng bèi nàgè bìngrén zhǐzé
has-DUR one-CL black hair REL doctor PAS that-CL patient accuse
That patient with black hair is accused by the doctor. (P)

(11) 穿著 綠色 上衣 的 小 男孩 追著 那個 女孩。
穿着 绿色 上衣 的 小 男孩 追着 那个 女孩。
chuānzhe lǜsè shàngyī de xiǎo nánhái zhuīzhe nàge nǚhái.
wear-DUR green shirt REL small boy chase-DUR that-CL girl
The little boy in the green shirt chases that girl. (A)

(12) 被 女人 錄影 的 那個 男人 有著 一頭 棕髮。
被 女人 录影 的 那个 男人 有着 一头 棕发。
bèi nǚrén lùyǐng de nàgè nánrén yǒuzhe yītóu zōngfà.
PAS woman record REL that-CL man has-DUR one-CL brown hair
That man that is recorded by the woman has brown hair. (bèi-OR)

(13) 推 小 男孩 的 那個 男人 穿著 紅色 上衣。
推 小 男孩 的 那个 男人 穿着 红色 上衣。
tuī xiǎo nánhái de nàge nánrén chuānzhe hóngsè shàngyī.
push small boy REL that-CL man wear-DUR red shirt
That man that pushes the little boy is wearing a red shirt. (SR)

(14) 男孩 抓著 的 那個 男人 有著 一頭 棕髮。

男孩 抓着 的 那个 男人 有着 一头 棕发。
nánhái zhuāzhe de nàge nánrén yǒuzhe yītóu zōngfà.
boy grab-DUR REL that-CL man has-DUR one-CL brown hair
That man that the boy grabs has brown hair. (OR)

(15) 被 小 男孩 指揮 的 那個 老師 穿著 藍色 上衣。
被 小 男孩 指挥 的 那个 老师 穿着 蓝色 上衣。
bèi xiǎo nánhái zhǐhuī de nàgè lǎoshī chuānzhe lánsè shàngyī.
PAS small boy instruct REL that-CL teacher wear-DUR blue shirt
That teacher that is instructed by the little boy is wearing a blue shirt. (bèi-OR)

(16) 有著 一頭 金髮 的 女孩 被 那個 護士 拍照。
有着 一头 金发 的 女孩 被 那个 护士 拍照。
yǒuzhe yītóu jīnfà de nǚhái bèi nàgè hùshì pāizhào.
has-DUR one-CL blonde hair REL girl PAS that-CL nurse photograph
The girl with blonde hair is photographed by that nurse. (P)

(17) 醫生 質問 的 那個 士兵 有著 一頭 金髮。
医生 质问 的 那个 士兵 有着 一头 金发。
yīshēng zhíwèn de nàge shìbīng yǒuzhe yītóu jīnfǎ.
doctor question REL that-CL soldier has one-CL blonde hair
That soldier that the doctor questions has blonde hair. (OR)

(18) 追著 女孩 的 那個 男孩 穿著 綠色 上衣。
追着 女孩 的 那个 男孩 穿着 绿色 上衣。
zhuīzhe nǚhái de nàge nánhái chuānzhe lǜsè shàngyī.
chase girl REL that-CL boy wear-DUR green shirt
That boy that chases the girl is wearing a green shirt. (SR)

(19) 有著 一頭 棕髮 的 男人 替 那個 女人 錄影。
有着 一头 棕发 的 男人 替 那个 女人 录影。
yǒuzhe yītóu zōngfà de nánrén tì nàgè nǚrén lùyǐng.
has-DUR one-CL brown hair REL man for that-CL woman record
The man with brown hair records that woman. (A)

(20) 園丁 抓到 的 那個 畫家 穿著 藍色 褲子。
园丁 抓到 的 那个 画家 穿着 蓝色 裤子。
yuándīng zhuādào de nàgè huàjiā chuānzhe lánsè kùzi.
gardener capture-arrive REL that-CL painter wear-DUR blue pants
That painter that the gardener captures has on blue pants. (OR)

(21) 穿著 藍色 上衣 的 老師 被 那個 男孩 指揮。
穿着 蓝色 上衣 的 老师 被 那个 男孩 指挥。
chuānzhe lánsè shàngyī de lǎoshī bèi nàgè nánhái zhǐhuī.
wear-DUR blue shirt REL teacher PAS that-CL boy instruct
The teacher wearing the blue shirt is instructed by that boy. (P)

(22) 有著 一頭 棕髮 的 男人 抓著 那個 男孩。
有着 一头 棕发 的 男人 抓着 那个 男孩。
yǒuzhe yītóu zōngfà de nánrén zhuāzhe nàge nánhái.
have-DUR one-CL brown hair REL man grab-DUR that-CL boy
The man with the brown hair grabs that boy. (A)

(23) 被 士兵 大吼 的 那個 小 男孩 有著 一頭 黑髮。
被 士兵 大吼 的 那个 小 男孩 有着 一头 黑发。
bèi shìbīng dàhǒu de nàgè xiǎo nánhái yǒuzhe yītóu hēifà.
PAS soldier yell REL that-CL small boy have-DUR one-CL black hair
That little boy that is yelled at by the soldier has black hair. (bèi-OR)

(24) 質問 醫生 的 那個 士兵 有著 一頭 金髮。
质问 医生 的 那个 士兵 有着 一头 金发。
zhíwèn yīshēng de nàge shìbīng yǒuzhe yītóu jīnfã.
question doctor REL that-CL soldier has-DUR one-CL blonde hair
That soldier that questions the doctor has blonde hair. (SR)

(25) 穿著 藍色 褲子 的 畫家 抓到 那個 園丁。
穿着 蓝色 裤子 的 画家 抓到 那个 园丁。
chuānzhe lánsè kùzi de huàjiā zhuādào nàgè yuándīng.
wear-DUR blue pants REL painter capture-arrive that-CL gardener
The painter with the blue pants captures that gardener. (A)

(26) 替 女人 錄影 的 那個 男人 有著 一頭 棕髮。
替 女人 录影 的 那个 男人 有着 一头 棕发
tì nǚrén lùyǐng de nàgè nánrén yǒuzhe yītóu zōngfà.
for girl record REL that-CL man has-DUR one-CL brown hair
That man that records the woman has brown hair. (SR)

(27) 被 女孩 追著 的 那個 男孩 穿著 綠色 上衣。
被 女孩 追着 的 那个 男孩 穿着 绿色 上衣。
bèi nǚhái zhuīzhe de nàge nánhái chuānzhe lǜsè shàngyī.
PAS girl chase REL that-CL boy wear-DUR green shirt
That boy that is chased by the girl is wearing a green shirt. (bèi-OR)

(28) 指責 病人 的 那個 醫生 有著 一頭 黑髮。
指责 病人 的 那个 医生 有着 一头 黑发。
zhǐzé bìngrén de nàgè yīshēng yǒuzhe yītóu hēifà.
accuse patient REL that-CL doctor has-DUR one-CL black hair
That doctor that accuses the patient has black hair. (SR)

(29) 有著 一頭 金髮 的 女孩 替 那個 護士 拍照。
有着 一头 金发 的 女孩 替 那个 护士 拍照。

yǒuzhe yītóu jīnfà de nǚhái tì nàgè hùshì pāizhào.
has-DUR one-CL blonde hair REL girl for that-CL nurse photograph
The girl with blonde hair photographs that nurse. (A)

(30) 被 醫生 質問 的 那個 士兵 有著 一頭 金髮。
被 医生 质问 的 那个 士兵 有着 一头 金发。
bèi yīshēng zhíwèn de nàge shìbīng yǒuzhe yītóu jīnfǎ.
PAS doctor question REL that-CL soldier has-DUR one-CL blonde hair
That soldier that is questioned by the doctor has blonde hair. (bèi-OR)

(31) 向 士兵 大吼 的 那個 小 男孩 有著 一頭 黑髮。
向 士兵 大吼 的 那个 小 男孩 有着 一头 黑发。
xiàng shìbīng dàhǒu de nàgè xiǎo nánhái yǒuzhe yītóu hēifà.
towards soldier yell REL that-CL little boy has-DUR one-CL black hair
That little boy that yelled at the soldier has black hair. (SR)

(32) 被 園丁 抓到 的 那個 畫家 穿著 藍色 褲子。
被 园丁 抓到 的 那个 画家 穿着 蓝色 裤子。
bèi yuándīng zhuādào de nàgè huàjiā chuānzhe lánsè kùzi.
PAS gardener capture-arrive REL that-CL painter wear-DUR blue pants
That painter that is captured by the gardener has on blue pants. (bèi-OR)

(33) 女人 錄影 的 那個 男人 有著 一頭 棕髮。
女人 录影 的 那个 男人 有着 一头 棕发。
nǚrén lùyǐng de nàgè nánrén yǒuzhe yītóu zōngfà.
woman record REL that-CL man has-DUR one-CL brown hair
That man that the woman records has brown hair. (OR)

(34) 穿著 綠色 上衣 的 男孩 被 那個 女孩 追著。
穿着 绿色 上衣 的 男孩 被 那个 女孩 追着。
chuānzhe lǜsè shàngyī de nánhái bèi nàge nǚhái zhuīzhe.
wear-DUR blue shirt REL boy PAS that-CL girl chase-DUR
The boy in the green shirt is chased by that girl. (P)

(35) 病人 指責 的 那個 醫生 有著 一頭 黑髮。
病人 指责 的 那个 医生 有着 一头 黑发。
bìngrén zhǐzé de nàgè yīshēng yǒuzhe yītóu hēifà.
patient accuse REL that-CL doctor has-DUR one-CL black hair
That doctor that the patient accuses has black hair. (OR)

(36) 抓到 園丁 的 那個 畫家 穿著 藍色 褲子。
抓到 园丁 的 那个 画家 穿着 蓝色 裤子。
zhuādào yuándīng de nàgè huàjiā chuānzhe lánsè kùzi.
capture-arrive gardener REL that-CL painter wear-DUR blue pants
That painter that captures the gardener has on blue pants. (SR)

(37) 穿著 紅色 上衣 的 男人 推 那個 小 男孩。
穿着 红色 上衣 的 男人 推 那个 小 男孩。
chuānzhe hóngsè shàngyī de nánrén tuī nàge xiăo nánhái.
wear-DUR red shirt REL man push that-CL little boy
The man in the red shirt pushes that little boy. (A)

(38) 有著 一頭 金髮 的 士兵 被 那個 醫生 質問。
有着 一头 金发 的 士兵 被 那个 医生 质问。
yǒuzhe yītóu jīnfǎ de shìbīng bèi nàge yīshēng zhíwèn.
have-DUR one-CL blonde hair REL soldier PAS that-CL doctor question
The soldier with blonde hair is questioned by that doctor. (P)

(39) 小 男孩 指揮 的 那個 老師 穿著 藍色 上衣。
小 男孩 指挥 的 那个 老师 穿着 蓝色 上衣。
xiǎo nánhái zhǐhuī de nàgè lǎoshī chuānzhe lánsè shàngyī
little boy instruct REL that-CL teacher wear-DUR blue shirt
That teacher that the little boy instructs is wearing a blue shirt. (OR)

(40) 拿著 書本 的 小 男孩 向 那個 士兵 大吼。
拿着 书本 的 小 男孩 向 那个 士兵 大吼。
názhe shūběn de xiǎo nánhái xiàng nàge shìbīng dàhǒu.
carry-DUR book-CL REL little boy towards that-CL soldier yell
The little boy with the book yells at that soldier. (A)

(41) 有著 一頭 棕髮 的 男人 被 那個 男孩 抓著。
有着 一头 棕发 的 男人 被 那个 男孩 抓着。
yǒuzhe yītóu zōngfà de nánrén bèi nàge nánhái zhuāzhe.
have-DUR one-CL brown hair REL man PAS that-CL boy grab-DUR
That man with the brown hair is grabbed by that boy. (P)

(42) 被 病人 指責 的 那個 醫生 有著 一頭 黑髮。
被 病人 指责 的 那个 医生 有着 一头 黑发。
bèi bìngrén zhǐzé de nàgè yīshēng yǒuzhe yītóu hēifà.
PAS patient accuse REL that-CL doctor has-DUR one-CL black hair
That doctor that is accused by the patient has black hair. (bèi-OR)

(43) 穿著 紅色 上衣 的 男人 被 那個 小 男孩 推。
穿着 红色 上衣 的 男人 被 那个 小 男孩 推。
chuānzhe hóngsè shàngyī de nánrén bèi nàge xiǎo nánhái tuī.
wear-DUR red shirt REL man PAS that-CL little boy push
The man in the red shirt is pushed by that little boy. (P)

(44) 護士 用 相機 拍 的 那個 女孩 有著 一頭 金髮。
护士 用 相机 拍 的 那个 女孩 有着 一头 金发。
hùshì yòng xiàngjī pāi de nàgè nǚhái yǒuzhe yītóu jīnfà.
nurse use camera photograph REL that-CL girl has-DUR one-CL blonde hair
That girl that the nurse photographs using the camera has blonde hair. (OR)

(45) 指揮 男孩 的 那個 老師 穿著 藍色 上衣。
指挥 男孩 的 那个 老师 穿着 蓝色 上衣。
zhǐhuī nánhái de nàgè lǎoshī chuānzhe lánsè shàngyī
instruct boy REL that-CL teacher wear-DUR blue shirt
That teacher that instructs the boy is wearing a blue shirt. (SR)

(46) 被 男孩 抓著 的 那個 男人 有著 一頭 棕髮。
被 男孩 抓着 的 那个 男人 有着 一头 棕发。
bèi nánhái zhuāzhe de nàge nánrén yǒuzhe yītóu zōngfà.
PAS boy grab-DUR REL that-CL man have-DUR one-CL brown hair
That man that is grabbed by the boy has brown hair. (bèi-OR)

(47) 有著 一頭 黑髮 的 男孩 被 那個 士兵 大吼。
有着 一头 黑发 的 男孩 被 那个 士兵 大吼。
yǒuzhe yītóu hēifà de nánhái bèi nàge shìbīng dàhǒu.
have-DUR one-CL black REL boy PAS that-CL soldier yell
The boy with black hair is yelled at by that soldier. (P)

(48) 小 男孩 推 的 那個 男人 穿著 紅色 上衣。
小 男孩 推 的 那个 男人 穿着 红色 上衣。
xiǎo nánhái tuī de nàge nánrén chuānzhe hóngsè shàngyī.
little boy push REL that-CL man wear-DUR red shirt
That man that the little boy pushes is wearing a red shirt. (OR)

(49) 穿著 藍色 上衣 的 老師 指揮 那個 小 男孩。
穿着 蓝色 上衣 的 老师 指挥 那个 小 男孩。
chuānzhe lánsè shàngyī de lǎoshī zhǐhuī nàgè xiǎo nánhái.
wear-DUR blue shirt REL teacher instruct that-CL little boy
The teacher wearing the blue shirt instructs that little boy. (A)

(50) 用 相機 拍 護士 的 那個 女孩 有著 一頭 金髮。
用 相机 拍 护士 的 那个 女孩 有着 一头 金发。
yòng xiàngjī pāi hùshì de nàgè nǚhái yǒuzhe yītóu jīnfà
use camera photograph nurse REL that-CL girl has-DUR one-CL blonde hair
That girl that photographs the nurse using the camera has blonde hair. (SR)
